# Cuproptosis-associated genes *DLD*, *ULK2*, *CCL8*, and *CCS* link metabolic and immunological dysfunction in Parkinson’s disease: a multi-omics study

**DOI:** 10.3389/fimmu.2026.1813166

**Published:** 2026-09-16

**Authors:** Jing Wang, Ya-Hui Hu, Bo Wang, Qi Liu, Yi-Dan Zhang, Kun-Mu Zheng, Nai-An Xiao, Li-Xi Li, Bin Jiang

**Affiliations:** 1 Department of Neurology and Department of Neuroscience, The First Affiliated Hospital of Xiamen University, School of Medicine, Xiamen University, Xiamen, China; 2 Xiamen Key Laboratory of Brain Center, Xiamen, China; 3 Xiamen Medical Quality Control Center for Neurology, Xiamen, China; 4 Department of Neurosurgery, The First Affiliated Hospital of Xiamen University, School of Medicine, Xiamen University, Xiamen, China; 5 Department of Radiology, Xiamen Cardiovascular Hospital of Xiamen University, School of Medicine, Xiamen University, Xiamen, China; 6 Department of Neurology, The Third Hospital of Xiamen, Xiamen, China; 7 Department of Neurology, Tongji Hospital, School of Medicine, Tongji University, Shanghai, China

**Keywords:** cuproptosis, multi-omics, multiplex immunofluorescence, Parkinson‘s disease, PD mouse model

## Abstract

Parkinson’s disease (PD) is a progressive neurodegenerative disorder whose pathogenesis involves complex interactions between metabolic dysfunction and neuroinflammation. The role of cuproptosis, a newly defined copper-dependent cell death pathway that is triggered during mitochondrial aerobic respiration, in PD remains incompletely understood. In this study, we employed integrated bioinformatics on bulk and single-cell RNA sequencing datasets to identify four hub cuproptosis-associated genes (*DLD*, *ULK2*, *CCL8*, and *CCS*) strongly linked to PD. Functional enrichment and immune infiltration analyses linked these genes to metabolic disturbances and altered immune landscapes in PD. Single-cell transcriptomics further revealed patterns of cell-type-specific expression of these genes and altered inferred intercellular communication, particularly through the NRG signaling pathway between microglia and astrocytes. Experimental validation in AAV9-A53T α-synuclein mouse models, utilizing cell-type-specific multiplex immunofluorescence (mIF) and western blot analysis, provided supportive evidence for reduced DLD levels in dopaminergic neurons, decreased ULK2 and CCL8 expression in microglia, and diminished CCS content in astrocytes, accompanied by increased FDX1 expression in these cell populations. These findings suggest that dysregulation of cuproptosis-associated hub genes is associated with cell-type-specific pathological alterations in PD, rather than demonstrating a direct causal or synergistic regulatory mechanism. Moreover, in silico drug–target interaction analysis identified four potential therapeutic compounds targeting these hub gene-related proteins. Our findings indicate a potential association between cuproptosis-associated gene dysregulation and metabolic–immune abnormalities in PD, providing candidate biomarkers and possible intervention targets for further validation.

## Introduction

Parkinson’s disease (PD) is the second most common neurodegenerative disorder globally after Alzheimer’s disease (1). It is characterized by motor impairments, including bradykinesia, resting tremor, muscle rigidity, and postural gait disturbances, leading to significant familial, medical, and socioeconomic burdens. Besides, non-motor symptoms of PD, including depression, sleep disorders, chronic pain, hyposmia, and autonomic dysfunction, are increasingly drawing global attention. Although many of these symptoms appear earlier than motor symptoms, they are often insidious and difficult to detect (2–4). While the exact cause of PD remains obscure, some studies have proposed close links among genetic factors, environmental factors, diet, age, and reactive oxygen stress (ROS) (5–7). Given the diverse manifestations of Parkinson’s disease symptoms and its complex etiology, there is an urgent need to identify novel biomarkers or characteristics to enhance early clinical decision-making in PD management.

The pathological hallmark of Parkinson’s disease is Lewy bodies which consist of abnormally aggregated intracellular α-synuclein and are associated with progressive loss of dopaminergic neurons (8, 9). Various factors involved in neuronal cell death mediate the progression of PD, including but not limited to mitochondrial malfunction, oxidative stress, autophagic dysfunction, apoptosis, necrosis, pyroptosis, ferroptosis, and neuroinflammation (2, 10, 11). Cuproptosis is a newly defined copper-dependent cell death pathway that depends on mitochondria respiration. It is characterized by the direct binding of copper ions, leading to the aggregation of lipoylated tricarboxylic acid (TCA) cycle proteins, which induces severe mitochondrial proteotoxic stress and metabolic collapse, unlike all other programmed cell death mechanisms (12). Ferredoxin-1 (*FDX1*) has been definitively established as a critical upstream modulator of cuproptosis. Its abnormal activation leads to the loss of Fe-S cluster proteins along with the aggregation of lipoylated proteins, subsequently triggering mitochondrial protein lipoylation (12, 13). Moreover, important roles for cuproptosis in the immune microenvironment, including neuroinflammation, have been unveiled (3, 14). Therefore, we hypothesize that cuproptosis is involved in the initiation and progression of Parkinson’s disease, not only directly inducing neuronal death, but also associated with mitochondria dysfunction and neuroinflammation, potentially serving as a therapeutic target for PD.

Our study systematically analyzed and validated the relationship between cuproptosis and PD. Through integrated expression profiles of three bulk mRNA datasets, we characterized 11 differentially expressed cuproptosis-associated genes (DECuAGs). Four hub DECuAGs related to the pathogenesis of PD were further identified by machine learning techniques and explored for their functional enrichment and immune infiltration. Subsequently, cell-specific expression patterns of these hub DECuAGs were validated using single-cell profiling analysis. Moreover, we verified these expression patterns utilizing mIF and Western blot in the successfully established AAV9-A53T α-synuclein PD mouse models. We also investigated the intercellular communications of the critical cells related to these hub genes. Finally, as an exploratory downstream translational extension, we performed DSigDB-based compound prediction, molecular docking, and molecular dynamics simulations to prioritize candidate compounds targeting proteins related to the hub DECuAGs for future validation. These discoveries may offer crucial perspectives for elucidating the diverse and intricate mechanisms underlying the pathogenesis of PD, thereby opening avenues for potential therapeutic strategies targeting cuproptosis-associated factors.

## Materials and methods

### Study design

The framework is shown in **Figure 1**.

**Figure 1:**
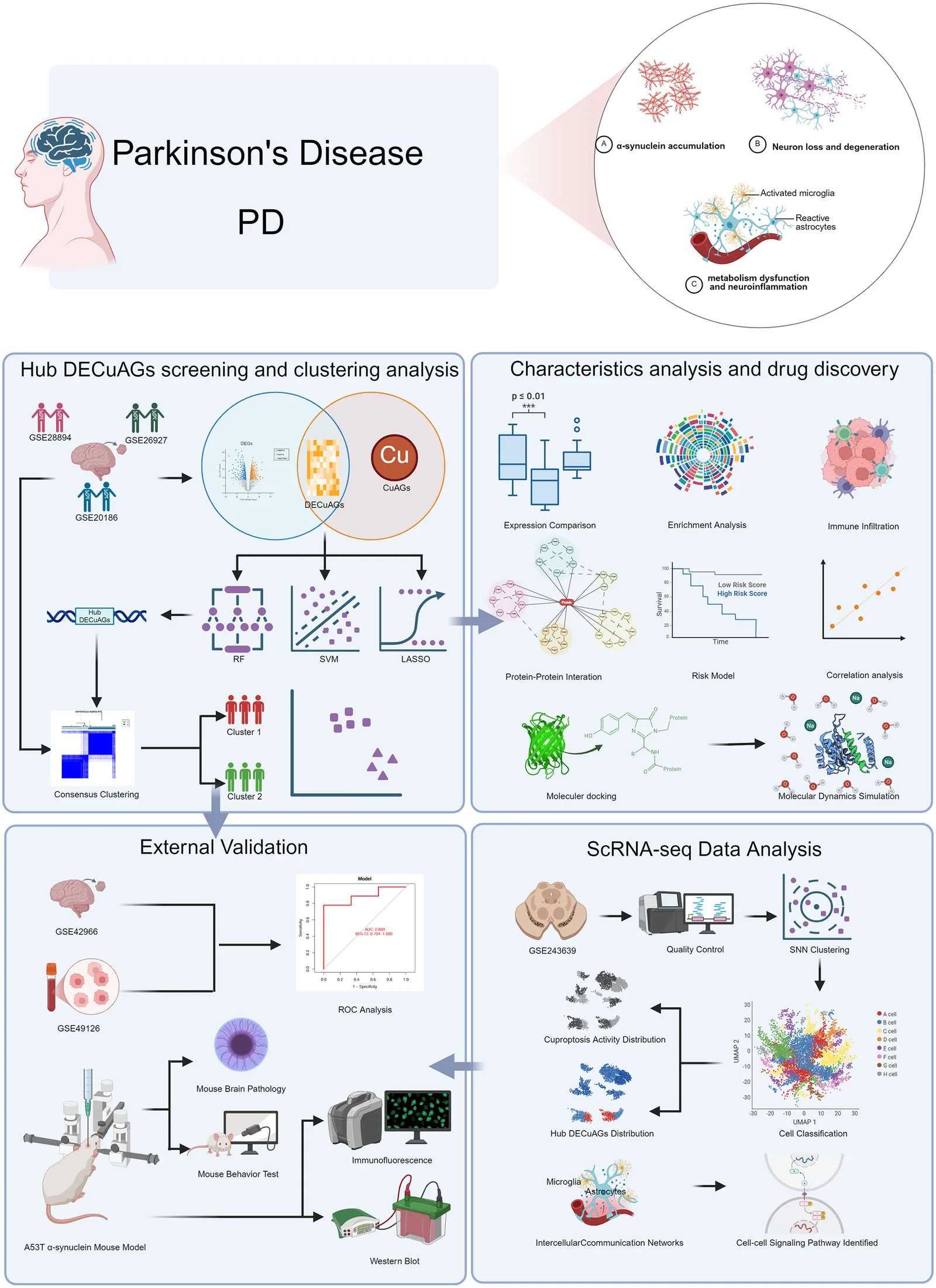
Research framework. Created by Biorender.com.

### Data acquisition and processing

In our study, we harvested five batch mRNA datasets (GSE28894, GSE26927, GSE20186, GSE42966, and GSE49126) and one single-cell RNA sequencing (scRNA-seq) dataset (GSE243639) from the Gene Expression Omnibus (GEO) database (http://www.ncbi.nlm.nih.gov/geo/), concerning PD patients and controls. Subsequent bulk data analyses used brain samples from a total of 85 PD patients and 85 matching control subjects (59 individuals with PD and 55 controls from GSE28894, 8 individuals with PD and 12 controls from GSE26927, 18 individuals with PD and 18 controls from GSE20186). GSE42966 and GSE49126 were used as external datasets for the hub-gene validation. The former comprised brain tissue samples from 9 PD patients and 6 controls, while the latter included blood samples from 30 PD patients and 20 controls. The scRNA-seq data analyses were processed on brain samples from 15 PD patients and 14 controls in the GSE243639 dataset. **Supplementary Table S1** provides detailed information about the datasets. Forty-six cuproptosis-associated genes (CuAGs) were extracted from the GeneCards database (https://www.genecards.org/) and prior publications (**Supplementary Table S2**). Data processing was performed using R software (v4.4.1). Each bulk mRNA dataset was normalized utilizing the normalizeBetweenArrays function in the “limma” package. We integrated normalized GSE28894, GSE26927, and GSE20186 datasets using the “sva” package, as well as removing the batch effects among different datasets.

### Differentially expressed CuAGs analysis

We performed differentially expressed genes (DEGs) analysis between Parkinson’s disease patients and controls on the previously merged dataset, utilizing the “limma” package. Genes meeting the criteria of |log2 fold change (FC) |> 1 and a Bonferroni-p-value < 0.05 were identified as differentially expressed. The “pheatmap” and “ggplot2” packages were used to visualize the DEGs. Functional annotation and pathway enrichment analysis of the DEGs were conducted using the Metascape (https://metascape.org/), Kyoto Encyclopedia of Genes and Genomes (KEGG), and Gene Ontology (GO) databases. We also employed the “clusterProfiler” and “fgsea” packages to compare changes in enriched biological pathways between PD and controls, considering p-values below 0.05 as indicative of significant alterations. By intersecting DEGs with 46 CuAGs, we further obtained DECuAGs and visualized them using the “ggpubr” package. A protein-protein interaction (PPI) network was then developed using the STRING online database (https://string-db.org/) to investigate the protein-level associations among these genes. Correlation analysis was performed using the “corrplot” and “circlize” packages. Afterward, these genes underwent GO, KEGG, Disease Ontology (DO), and Metascape analyses to identify key biological terms. Finally, we exploited the GeneMANIA online database (https://genemania.org) to pinpoint potentially relevant genes for these DECuAGs.

### Hub DECuAGs screening and validation

To further characterize the signature genes of PD, we performed multiple machine-learning algorithms on the expression profiles of 11 DECuAGs. First, we randomly divided the merged dataset into a training set (70%, PD = 57, Control = 62) and a test set (30%, PD = 28, Control = 23). Next, we employ the “glmnet” package to perform univariate logistic regression analysis, thereby narrowing down the list of signature genes as a starting point for constructing machine learning models (15). We then employed the “glmnet”, “e1071”, and “randomForest” packages to screen for the PD-related DECuAGs using the Least Absolute Shrinkage and Selection Operator (LASSO), Support Vector Machines Recursive Feature Elimination (SVM-RFE), and Random Forest (RF) algorithms, respectively. LASSO adds regularization terms into the loss function to improve the model’s prediction accuracy and explainability. We performed LASSO regression analysis with 10-fold cross-validation to find the best lambda and to discourage overfitting (16). SVM-RFE locates the optimum variable by finding the match with the minimum cross-validation error to identify the most discriminating genes. We iteratively trained the SVM-RFE model with 10-fold cross-validation to gradually reduce the feature set (17). RF algorithm aggregates variable importance measures from numerous decorrelated decision trees to identify the most relevant variables. We constructed 500 decision trees by Bootstrap sampling and quantified gene importance to systematically select the most influential feature genes (top 10 in empirical value) (18). The overlapping results of the three algorithms were considered hub genes that contribute to cuproptosis activity in PD patients with high robustness and confidence. On the basis of the transcription profiles from the test cohort, receiver operating characteristic (ROC) analysis was performed to evaluate the diagnostic accuracy of PD for each hub DECuAG and logistic model. Subsequently, the R package “rms” was used to construct a nomogram for forecasting PD risk. Each hub DECuAG was assigned a corresponding score on the Points scale, and the Total Points were then converted to the predicted probability of PD. The predictive performance and robustness of the nomogram were evaluated by a calibration curve and decision curve analysis (DCA). Finally, we validate the risk model with ROC curves using GSE42966 and GSE49126 as external datasets.

### Hub DECuAGs characteristics and unsupervised clustering

The previously identified hub DECuAGs were visually processed by the “ggpubr” package. The location map for the hub DECuAGs in 23 chromosomes was generated using the “RCircos” package. The correlations between these genes were displayed using the “corrplot” package. We employed gene set enrichment analysis (GSEA) to investigate the enriched signaling pathways for the four hub genes in PD, with a P-value below 0.05 regarded as statistically significant. To evaluate the activity of specific biological pathways in each sample, single-sample gene set enrichment analysis (ssGSEA) was performed. Spearman’s rank correlation tests were conducted between enriched HALLMARK pathways and the four hub genes. Unsupervised clustering of these genes in previous PD data was then performed with the “ConsensusClusterPlus” R package. The algorithm included 50 random subsampling operations, each selecting 80% of the samples. The optimal clustering number was determined by considering the features of consensus matrix heatmaps, the clustering score (> 0.9), and the cumulative distribution function (CDF) curves. The distribution of samples was visualized using principal component analysis. Differences in gene expression and immune-infiltrating cells were investigated across the various clusters. Additionally, gene set variation analysis (GSVA) was employed to investigate the variation in enriched biological processes within each cluster. The referenced datasets “c5.go.symbols” and “c2.cp.kegg.symbols” were both obtained via the MSigDB repository (https://www.gsea-msigdb.org/).

### Immune characteristics

CIBERSORT is a computational tool based on bulk RNA sequencing datasets that assesses the composition ratio of different cell subpopulations in organisms. The reference gene expression feature matrix used for deconvolution comprises 547 biosignatures capable of distinguishing 22 human immune cell phenotypes. We employed it to analyze the merged dataset in order to investigate the immuno-microenvironment in PD patients. A sample with a p-value less than 0.05 is considered statistically significant. Subsequently, we used the “ggpubr” package to display the ratio of different immune cell types in control and PD samples. Ultimately, the relationships between the hub DECuAGs and the infiltrating immunocytes in PD samples were examined via the Spearman’s rank correlation test and demonstrated with the “ggplot2” and the “linkET” packages.

### Single-cell analysis

We processed GSE243639 for single-cell analysis using the “Seurat” package (v5.0.1). First, quality control was performed on this dataset, and genes/features or cells matching the criteria below were excluded: 1) genes/features shared by fewer than 3 cells; 2) cells containing fewer than 500 genes/features with greater than 0 expression; and 3) cells with mitochondrial ratio exceeding 10%. Subsequently, the preprocessed data were standardized through the “NormalizeData” function to eliminate technical variation while preserving biological differences. The “FindVariableFeatures” module was then employed to pinpoint the 3,000 genes exhibiting the highest degree of variation. For data centering and scaling, the “ScaleData” function was used. Principal component analysis (PCA) was then performed to reduce the number of data dimensions and extract key sources of variation. The “RunHarmony” function was implemented to remove artificial variation caused by batch effects. Next, we constructed a shared nearest-neighbor (SNN) graph for clustering analysis and employed the Louvain/Leiden algorithm at a resolution of 0.02 to identify cellular clusters. The outcomes were further displayed via the Uniform Manifold Approximation and Projection (UMAP) method. We used the “FindAllMarkers” function to identify DEGs for each cluster and filtered out genes expressed in over 25% of cells within each cluster. Cell identity assignment was done automatically by the “SingleR” function and further verified manually. The canonical marker genes for human brain cells were identified using the CellMarker 2.0 repository (https://bio-bigdata.hrbmu.edu.cn/CellMarker/). Cell composition was analyzed using the OmicStudio tools at https://www.omicstudio.cn. Finally, to achieve statistical equivalence, we randomly downsampled the data for approximately 5000 cells each and represented the results with a density plot of UMAP.

### Evaluation of cuproptosis activity and the cuproptosis-associated hub genes expression in single-cell data

To better understand the signature of cuproptosis in PD, we employed the “GetAssayData” algorithm to assess the average expression of cuproptosis-related genes (**Supplementary Table S2**) as cuproptosis activity scores. The results were visualized through UMAP analysis. Subsequently, we validated the differential expression of the aforementioned 4 cuproptosis-associated hub genes and the pivotal cuproptosis gene *FDX1* within the identified cell types using volcano plots. At last, their expression levels were assessed and visualized through density plots and violin plots.

### Cell-type-specific in silico perturbation analysis

To further explore the potential functional significance of the cell-type-specific expression of the four hub DECuAGs, we performed cell-type-specific in silico perturbation analyses using scTenifoldKnk for virtual gene knockout (19) and a scTenifoldNet-based in silico gene overexpression workflow (termed scTenifoldOE in this study) (20). Simulated knockout and overexpression analyses were performed independently in the corresponding cell populations under both control and PD conditions. The top 5000 highly variable genes, together with the target gene, were selected to construct the input expression matrix. For each perturbation analysis, 10 gene regulatory sub-networks were generated using 500 randomly selected cells. Genes with an adjusted *P* value < 0.05 were considered significantly responsive to the simulated perturbation. GO and KEGG enrichment analyses of the significantly responsive genes were subsequently performed using the clusterProfiler package.

### Intercellular communication and signal transduction pathway analysis

CellChat (version 1.6.1) serves as a tool designed to infer, analyze, and visualize interactions among ligands, receptors, and their cofactors (21). Thus, we used it to characterize the key signals and to build cell-cell communication networks among identified cell types. The “computeCommunProb” algorithm was used to infer potential intercellular communications. The “filterCommunication” function was used to filter interactions involving fewer than 10 cells. We utilized the “computeCommunProbPathway” module to further speculate on pathway-level interaction networks. Subsequently, the “aggregateNet” algorithm was performed to quantitatively analyze the amount of ligand-receptor and the strength of intercellular communication among different cell populations. Finally, the “NetAnalysis_” and “NetVisual_” functions were employed to represent signal transduction pathways of interest.

### Mice

All animal experiments were conducted in accordance with the National Institutes of Health Guide for the Care and Use of Laboratory Animals and were approved by the Ethics Committee of Tongji University (Approval No. TJBC00325107). The Adeno-associated Vector serotype 9 expressing human mutated A53T-α-synuclein (AAV9-A53T) and the Adeno-associated Vector serotype 9 expressing green fluorescent protein (AAV9-GFP) vectors were designed by WZ Biotechnology (Shandong, China). A total of 48 12-week-old wild-type C57BL/6 male mice were used in the animal validation experiments. Among them, 18 mice were used for the initial model validation and expression analysis cohort, and 30 mice were used for the pharmacological modulation cohort. In the initial model validation and expression analysis cohort, mice were randomly assigned to either the Control group or the PD group (n = 9 per group). In the pharmacological modulation cohort, mice were randomly assigned to six groups: Control + Vehicle, Control + tetrathiomolybdate (TTM), Control + Elesclomol-Cu, PD + Vehicle, PD + TTM, and PD + Elesclomol-Cu (n = 5 per group). Detailed drug and vehicle administration protocols are described in the section “Pharmacological modulation of cuproptosis-related molecular activity”. Mice under deep anesthesia (intraperitoneal injection of 0.5% sodium pentobarbital at a dose of 10 mL/kg) received unilateral injections into the right substantia nigra (SN). According to the mouse brain atlas of Paxinos and Franklin, the injection coordinates relative to the Bregma point were: anterior-posterior (AP) -3.0 mm, medial-lateral (ML) -1.3 mm, dorsal-ventral (DV) -4.5 mm. Concentrations of 1×10¹³ vg/mL AAV9-A53T or AAV9-GFP vectors were delivered using a microinjector at a speed of 0.1 μL/min (total dose: 1.0 μL). Mice in the PD or PD-related groups (PD, PD + TTM, and PD + Elesclomol-Cu) were injected with AAV9-A53T, whereas mice in the Control or Control-related groups (Control, Control + TTM, and Control + Elesclomol-Cu) were injected with AAV9-GFP. The condition of mice was continuously monitored for several hours to days after surgery to ensure recovery from the incision site. To validate the success of model establishment, one mouse brain from each group in the initial model validation and expression analysis cohort was embedded and sliced coronally at 30-μm thickness using a cryostat microtome for immunofluorescent double staining of tyrosine hydroxylase (TH, a marker for dopaminergic neurons) and α-synuclein (aSyn). The SN and caudate nucleus-putamen (CPU) sections were blocked and permeabilized for 1 hour at room temperature in a solution containing 5% bovine serum albumin (BSA) and 0.3% Triton X-100. They were then incubated at 4 °C overnight using anti-TH antibody (1:1000, ab6211, Abcam) and anti-aSyn antibody (1:200, MA5-12272, Invitrogen). It was then incubated with the fluorescently labeled secondary antibodies for 2 h at room temperature. Image acquisition was performed using a fluorescence microscope (Leica DM6B).

For the initial model validation and expression analysis cohort, following the behavioral testing (12 weeks after AAV9 injection), brains were immediately harvested from five mice per group for subsequent Western blot assays. The remaining mice per group were prepared for immunofluorescence staining as described below. For the pharmacological modulation cohort, after completion of the pharmacological intervention period at 15 weeks after AAV9 injection, ipsilateral SN tissues were rapidly collected from five mice per group for subsequent Western blot assays. For immunofluorescence staining, mice were deeply anesthetized with intraperitoneal 1% sodium pentobarbital at 15 mL/kg. Adequate anesthetic depth was confirmed by the absence of a pedal withdrawal reflex in response to toe pinch before cardiac perfusion was initiated. The mice were then transcardially perfused with 1× phosphate-buffered saline (PBS), followed by 4% formaldehyde solution. The brains were subsequently removed and immersed in 4% formaldehyde for 24 h. Post-fixed brains were cryoprotected by dehydration through sequential immersion in sucrose gradient solutions, followed by embedding in optimal cutting temperature (OCT) medium (Sakura Finetek, USA) and stored at -80 °C.

### Behavioral studies

Behavioral studies (open field test, rotarod test, and pole test) were conducted in the initial model validation and expression analysis cohort at 12 weeks after AAV9 injection (22, 23).

During the open field test, mice and their cage were placed in an opaque resin glass chamber measuring 40cm × 40cm × 30cm for a 30-minute acclimatization period. They were then released from the cage into the center of the chamber to roam freely for 15 minutes, with the entire procedure recorded via video surveillance. Total movement distance of mice around the peripheral and central regions of the chamber was tracked using SMART (v3.0) software.

In the rotarod test, mice were acclimated to a rotating apparatus for one day with a consistent rotating speed of four revolutions per minute (rpm). Then, mice were placed on the apparatus, which ramped up from 4 rpm to 40 rpm over a 5-minute period. Each experiment was repeated three times, with trial intervals exceeding thirty minutes, and the average fall latency was recorded.

For the pole test, mice were conditioned to descend a rough-surfaced upright pole spontaneously (height: 50 cm; diameter: 0.5 cm). The time required for mice to descend from the top was recorded. Each mouse underwent 3 independent tests to determine the average value, with trial intervals exceeding fifteen minutes.

### Multiplex immunofluorescence staining and measurement of fluorescence intensity

To observe the co-localization of hub DECuAGs with their corresponding brain cells (DLD- dopaminergic neurons, CCS- astrocytes, and ULK2, CCL8- microglia), as well as intracellular cuproptosis activity (colocalized with FDX1), we employed a quadruple-color Fluorescence kit (Hunan AiFang Biotechnology Co., Ltd.) for multiplex immunofluorescence (mIF). The kit utilized tyramide signal amplification technology, with all operational steps strictly adhering to the manufacturer’s instructions. Brain slices were obtained from 6 mice in the initial model validation and expression analysis cohort (n = 3 per group) using the procedure as described above. Detailed information on the antibodies and their dilution ratios is as follows: anti-DLD (1:500, sc-365977, Santa Cruz Biotechnology), anti-ULK2 (1:500, GTX111476, GeneTex), anti-CCS (1:500, sc-55561, Santa Cruz Biotechnology), anti-CCL8 (1:400, bs-1985R, Bioss Antibodies), anti-FDX1 (1:200, R014408, Yamei Biotechnology), anti-IBA1 (1:3000, AFRM0011, AiFang biological), anti-GFAP (1:3000, AFRM0041, AiFang biological), anti-TH (1:500, AFRM0092, AiFang biological).

To quantify mIF results, three random fields of view were captured from each sample. Images were acquired at ×50 magnification (for DaNs observation) or ×30 magnification (for ASTs and MGs observation) and used for positive staining analysis. After background subtraction, the fluorescence intensity of each target protein was quantified using ImageJ software (National Institutes of Health, Bethesda) and normalized to the number of corresponding marker-positive cells in each field. The mean value of the three fields was used for each sample and expressed relative to the control group.

### Pharmacological modulation of cuproptosis-related molecular activity

To further examine whether cuproptosis-related molecular alterations and dopaminergic neuronal marker changes were responsive to pharmacological modulation, intervention was performed from 12 to 15 weeks after AAV9 injection. To minimize the potential effects of administration route, handling, and vehicle exposure, all groups received route-matched vehicle or active drug treatment during the intervention period. Accordingly, the six groups were Control + Vehicle, Control + TTM, Control + Elesclomol-Cu, PD + Vehicle, PD + TTM, and PD + Elesclomol-Cu. The Control + Vehicle and PD + Vehicle groups received oral vehicle once daily and intraperitoneal vehicle five times per week. Mice in the Control + TTM and PD + TTM groups received TTM by oral gavage once daily and intraperitoneal vehicle five times per week. Mice in the Control + Elesclomol-Cu and PD + Elesclomol-Cu groups received oral vehicle once daily and Elesclomol-Cu by intraperitoneal injection five times per week. Vehicle solutions were administered at the same volume and schedule as the corresponding active drug treatments.

Tetrathiomolybdate (TTM; HY-128530, MedChemExpress) was used as a copper chelator to suppress copper-dependent cuproptosis-related molecular activity. TTM was freshly dissolved in sterile water and administered by oral gavage at a dose of 30 mg/kg/day once daily for 3 consecutive weeks (24). The corresponding oral vehicle was sterile water. Elesclomol-Cu was used as a copper ionophore-based activator of cuproptosis-related molecular activity (12). For Elesclomol-Cu treatment, elesclomol (HY-12040, MedChemExpress) was co-administered with cupric chloride (CuCl_2_; HY-107972, MedChemExpress) at doses of 40 mg/kg and 0.06 mg/kg, respectively, by intraperitoneal injection five times per week for 3 consecutive weeks (25). The Elesclomol-Cu working solution was freshly prepared on the day of administration in an intraperitoneal vehicle consisting of 10% DMSO, 40% PEG300, 5% Tween-80, and 45% 0.9% sterile saline. The corresponding intraperitoneal vehicle contained the same solvent system without elesclomol or CuCl_2_.

At 15 weeks after AAV9 injection, ipsilateral SN tissues were rapidly collected from five mice per group for Western blot analysis. This pharmacological modulation experiment was designed to evaluate the responsiveness of FDX1, hub DECuAG proteins, and TH to pharmacological modulation of cuproptosis-related molecular activity, rather than to establish gene-specific gain- or loss-of-function effects.

### Western blot analysis

The Western blot sample preparation method is as described previously (26). Stored tissue samples collected as described above were kept at -80 °C until use, and each sample was thawed only once. Tissue lysates prepared from these samples were subjected to sodium dodecyl sulphate-polyacrylamide gel electrophoresis (SDS-PAGE) and blotted onto polyvinylidene fluoride membranes (Millipore, Billerica, USA). Membranes were overnight incubated at 4°C using primary antibodies specific to DLD (1:1000, sc-365977, Santa Cruz Biotechnology), ULK2 (1:500, GTX111476, GeneTex), CCS (1:1000, sc-55561, Santa Cruz Biotechnology), CCL8 (1:1000, bs-1985R, Bioss Antibodies), FDX1 (1:3000, R014408, Yamei Biotechnology), TH (1:2000, AFRM0092, AiFang Biotechnology), and β-actin (1:4000, 20536-1-AP, Proteintech), then incubated with horseradish peroxidase-labeled anti-rabbit or anti-mouse antibodies (1:5000, HS101–01 or HS201, TransGen). Chemiluminescent signals were detected and captured via a JS-1070 imager (Peiqing Science & Technology, Shanghai, CN). Finally, band intensities were quantified using ImageJ after background subtraction, and the intensity of each target protein was normalized to the β-actin intensity in the corresponding lane.

### Drug screening and molecular docking

Following the identification of hub cuproptosis-associated genes implicated in Parkinson’s disease, we sought to determine potential drugs capable of modulating the expression of these genes to treat PD. The Drug Signature Database (DSigDB) (https://dsigdb.tanlab.org/DSigDBv1.0/) is a repository linking drugs to their induced gene expression alterations. It serves as a valuable resource for drug characterization analysis, aiding in the identification of putative drug-gene interactions (27). The drugs exhibiting the highest enrichment scores for each of the hub DECuAGs-related proteins within this database were selected as ligands for subsequent molecular docking experiments. Subsequently, we validated the intermolecular interactions between the chosen drugs and their protein targets through molecular docking analysis. This computational simulation method achieves this objective by providing a detailed analysis of their binding patterns and affinities (28). The process was carried out in the following steps: the three-dimensional atomic structures of the chosen drugs and their protein targets were downloaded from the PubChem database (https://pubchem.ncbi.nlm.nih.gov/) and the RCSB Protein Data Bank (PDB) (https://www.rcsb.org/). Molecular docking analysis was conducted for each protein-drug pair using an online automated docking tool (https://cadd.labshare.cn/cb-dock2/php/index.php). Five docking complexes were obtained from each analysis. Complexes with the most optimal (lowest) docking score were considered the most stable predicted conformations, with their protein-ligand interactions visualized. They were subsequently served as initial structures for molecular dynamics simulations.

### Molecular dynamics simulations

MD simulations constitute a computational method focused on the behavior of atoms and molecules at the microscopic scale, aiming to elucidate the dynamic details, stability, and interaction mechanisms of protein-drug binding processes (29). Each target protein-drug complex MD simulation was carried out using the YASARA Dynamics software (YASARA Biosciences, Vienna, Austria) with the AMBER14 force field. Every complex was prepared by optimizing the hydrogen-bond network and solvated using the TIP3P water model within a simulated unit cell under periodic boundary conditions. The energy minimization of the system was achieved through simulated annealing with a steepest gradient descent method of 5000 iterations. A repeated time-step method was employed for each simulation under physiological conditions of 298 K and pH 7.4, with a time step interval set at 1.25 fs. Bond lengths were constrained using the LINCS algorithm, and the SETTLE algorithm was used for water geometry. The root-mean-square deviation (RMSD) and molecular mechanics Poisson-Boltzmann surface area (MM-PBSA) binding free energy were calculated to estimate conformational stability and quantitatively assess the protein-ligand binding affinity. The analyses were performed using the default script of the YASARA macro and visualized via GraphPad Prism 9.5 software (GraphPad Software, Boston, USA).

### Statistics

Bioinformatics analyses were performed using relevant software packages in R (version 4.4.1), while *in vivo* experimental data were analyzed using GraphPad Prism 9.5 software. Spearman’s correlation analysis was used to determine the correlation. For continuous data following a normal distribution, the parametric Student’s t-test was used to perform comparisons; otherwise, the Mann-Whitney U test was employed. For pharmacological modulation experiments, data were analyzed by two-way ANOVA followed by Šídák’s multiple-comparisons test for pre-specified pairwise comparisons. Data were presented as mean ± standard error of the mean (SEM). Statistical significance was determined using a two-tailed p-value < 0.05 (***p < 0.001, **p < 0.01, *p < 0.05).

## Results

### Brain expression profiling analysis of PD patients

We analyzed three brain expression profiles that included a total of 85 PD patients and 85 matching controls (**Figure 2A**). The datasets were merged, and batch effects were eliminated (**Figure 2B**). We identified 1487 DEGs in the merged dataset, including 793 downregulated genes and 694 upregulated genes (**Figures 2C, D**; **Supplementary Table S3**). Subsequently, we analyzed these DEGs using the Metascape database to investigate their putative functional roles. The terms were observed highly related to “carboxylic acid metabolic process” and “metabolism of amino acids and derivatives”, which were strongly associated with biological metabolism (**Figure 2E**; **Supplementary Figure S1A**). Coincidentally, GO enrichment analysis highlighted a significant enrichment of terms related to “amino acid catabolic process” within the biological process module. As for modules of cellular component and molecular function, enrichment of these genes was detected in “mitochondrial protein- containing complex” and “organic acid binding” (**Supplementary Figures S1B, C**; **Supplementary Table S4**). Similarly, pathway enrichment analysis using KEGG also demonstrated predominantly enrichment in “Citrate cycle (TCA cycle)”, “Pyruvate metabolism”, and “lipoic acid metabolism” (**Supplementary Figures S1D, E**; **Supplementary Table S4**). In addition, we conducted GSEA analyses on the merged data and found that pathways closely related to mitochondrial energy metabolism were highly enriched in PD patients (**Figure 2F**; **Supplementary Table S5**).

**Figure 2:**
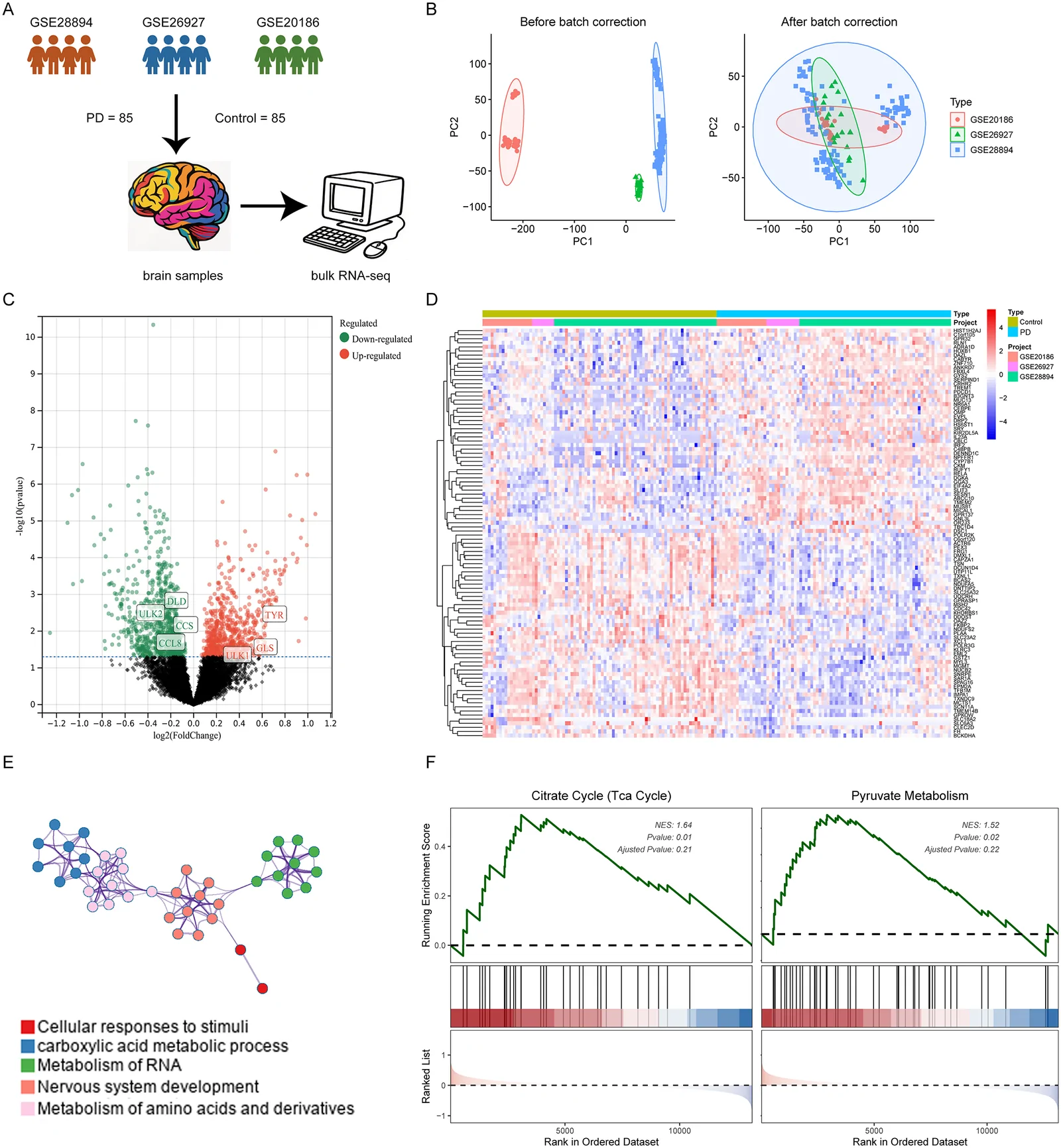
Analysis of DEGs between PD and the controls. **(A)** The GEO datasets and sample size used in this study are demonstrated in the diagram. **(B)** Post-processing of the integrated GEO datasets. **(C)** Volcano plot showing down-/up-regulated DEGs between PD and the controls. **(D)** Heatmap of DEGs between PD and the controls (top 50). **(E)** Network of biological function and pathway enrichment using the Metascape database (top 5). **(F)** GSEA analysis of metabolic pathways enriched in PD.

### DECuAGs analysis of PD patients

Thus, we focused on the cuproptosis and explored the pattern of the cuproptosis-associated gene expression in PD. By intersecting 1487 DEGs with these genes, eleven DECuAGs were screened from the merged dataset. A protein-protein interaction (PPI) network was then generated from these genes, after setting a medium confidence level, which vividly showed the genes and their close protein interactions (**Figure 3A**). Among these, *TYR*, *ULK1*, and *GLS* were upregulated in the PD group, while *CCS*, *SLC25A3*, *ULK2*, *CCL8*, *DLD, DLAT*, *LIAS*, and *PDHA1* were downregulated in the PD group (**Figure 3B**). A gene relationship network then indicated the tight association among these DECuAGs (**Figure 3C**). We also performed correlation analysis, revealing the strong interactions of these DECuAGs (**Figure 3D**). Notably, *DLD* was positively correlated with *SLC25A3*, *PDHA1*, and *DLAT*. Similarly, *DLAT* was positively correlated with *SLC25A3* and *PDHA1*. *LIAS* was positively related to *SLC25A3*, *DLAT*, and *CCS*. *ULK2* was negatively associated with *ULK1* and *GLS*. Coincidentally, *CCL8* was negatively associated with *ULK1* and *GLS*, and it was positively related to *ULK2*. *ULK1* was significantly positively correlated with *GLS*. Afterward, functional enrichment analysis on GO terms and KEGG pathways of these genes were conducted. We observed significantly enriched GO terms including the “acetyl-CoA biosynthetic process from pyruvate” (BP), “mitochondrial matrix” (CC), and “copper ion binding” (MF) (**Supplementary Figures S2A, B**; **Supplementary Table S6**). Correspondingly, pathways related to “Lipoic acid metabolism”, “Citrate cycle (TCA cycle)”, and “Pyruvate metabolism” were significantly enriched in KEGG analysis. It is notable that the “Pathways of neurodegeneration - multiple diseases” was also significantly enriched, which was consistent with the characteristics of the data (**Figures 3E, F**; **Supplementary Table S6**). **Figure 3G** demonstrated the combination of 20 predicted genes with 11 DECuAGs involved in several biological processes, including “acyl-CoA metabolic process”, “acyl-CoA biosynthetic process”, and “tricarboxylic acid cycle enzyme complex”. Finally, we conducted disease enrichment analysis via the Metascape platform and employed the DO analysis method to investigate the relationships of these genes with diseases. Our findings revealed that these genes exhibit significant associations with metabolic disorders, such as “lactic acidosis”, “metabolic acidosis”, “Pyruvate Dehydrogenase Complex Deficiency Disease”, “Increased serum lactate”, and “Acidosis, Lactic” (**Supplementary Figures S2C, D**; **Supplementary Table S6**).

**Figure 3:**
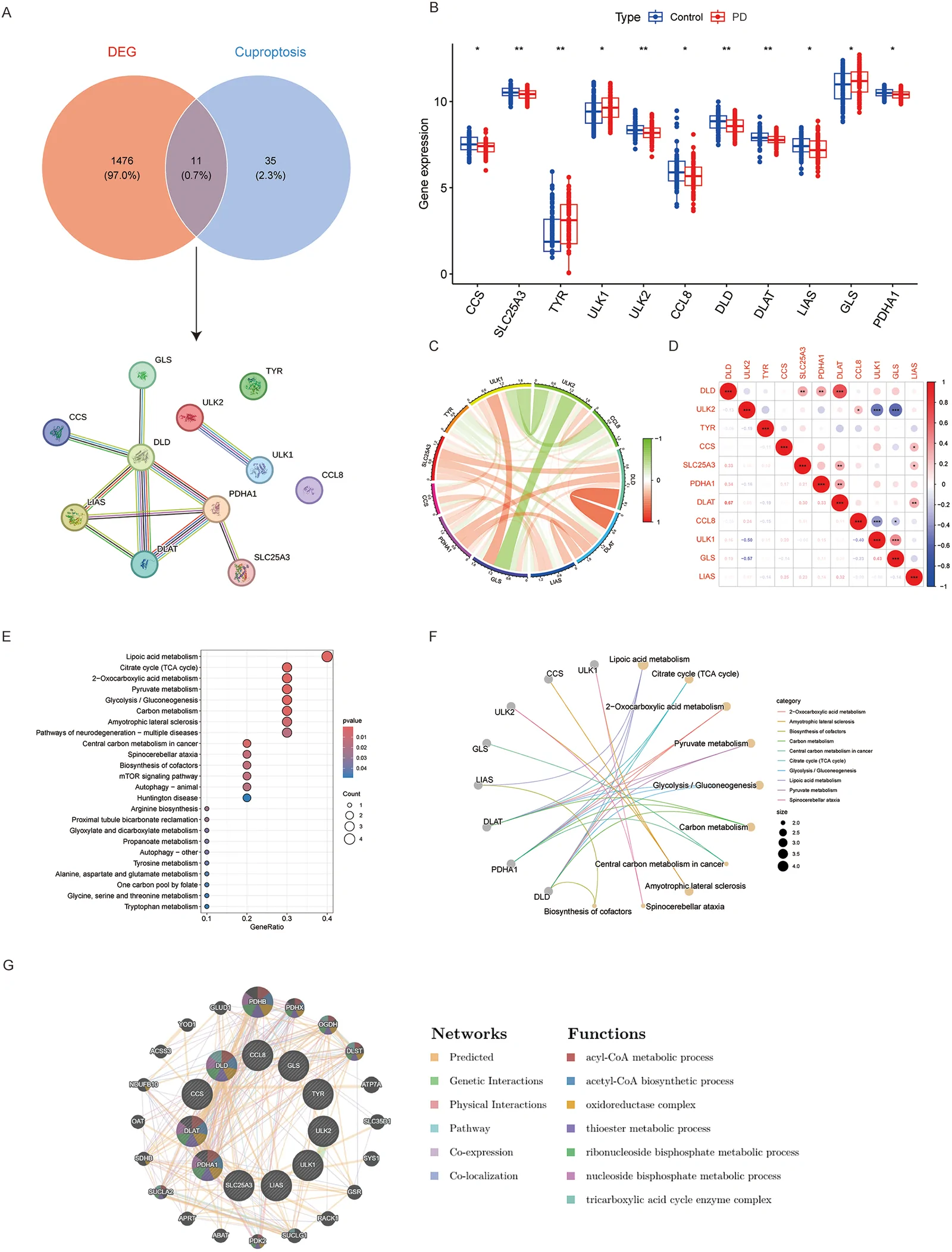
Identification of DECuAGs between PD and the controls. **(A)** Venn plot depicting DECuAGs by overlaying DEGs with CuAGs, and their PPI network constructed by plotting interaction scores with medium confidence (>0.400). **(B)** Box plot comparing the mRNA expression levels of DECuAGs between PD and the controls. **(C)** Ring plot of the gene relationship network for DECuAGs. Red bands represent positive correlations, while green bands represent negative correlations. **(D)** Correlation heatmap of the DECuAGs. Values and color scale represent Pearson correlation coefficients. **(E)** Bubble plot of enriched KEGG pathways in DECuAGs. The bubble size represents the number of genes (Count), and the color corresponds to P values. **(F)** Gene-concept network revealing the KEGG terms enriched in DECuAGs. Nodes represent genes (grey) and biological pathways (colored according to legend). **(G)** GeneMANIA interaction network for DECuAGs and their potentially associated genes. Edges between nodes (genes) are colored according to interaction type, while node colors represent primary biological functions. *P < 0.05, **P < 0.01, and ***P < 0.001.

### Identification of hub DECuAGs and construction of the risk model

To identify DECuAGs associated with Parkinson’s disease, we first performed one-way logistic regression analysis on the previously found 11 DECuAGs in the training cohort. All 11 DECuAGs were found to be strongly linked to PD (**Supplementary Table S7**). Subsequently, we conducted three machine learning techniques on the obtained significant logistic regression gene expression profiles to further screen for the feature genes in PD. By overlapping the results acquired from LASSO regression (**Figures 4A, B**), SVM algorithm (**Figures 4C, D**), and Random Forest model (**Figure 4E**), four genes that might be strongly associated with the occurrence of PD, including *DLD*, *CCL8*, *CCS*, and *ULK2*, were ultimately identified as hub DECuAGs in our subsequent research (**Figure 4F**). We then performed ROC analyses on the test cohort to assess the diagnostic efficiency of each hub DECuAG and the overall model. **Figures 4G, H** demonstrated the area under the curve (AUC) values for the model and individual biomarkers: 0.824 for *DLD*, 0.905 for *ULK2*, 0.756 for *CCS*, 0.687 for *CCL8*, and 0.944 (95% CI: 0.885-1.000) for the whole model, which suggested that the overall model performed most satisfactorily in identifying patients with PD. Therefore, we created a nomogram and selected all four hub DECuAGs as the variables predicting the risk of PD. Each gene was assigned a score on the points scale. The sum of these individual scores yielded the total points. When the total points were ≤100, the predicted probability of PD was below 0.1; when the total points were >230, this probability exceeded 0.99 (**Figure 4I**). The calibration plot demonstrated strong agreement between observed results and the predicted PD probabilities in the nomogram (**Figure 4J**). The DCA indicated the risk model provided promising predictive effectiveness and helped clinicians decide on therapeutic strategies (**Figure 4K**). In addition, we employed two human PD datasets, including a brain tissue dataset (GSE42966) and a blood sample dataset (GSE49126), to validate the risk model. The brain tissue dataset showed a higher validation performance, with an overall AUC value of 0.889 (95% CI: 0.704–1.000), whereas the blood sample dataset showed only modest performance, with an AUC value of 0.690 (95% CI: 0.525–0.838) (**Figures 4L, M**). This difference suggests that the hub DECuAG signature may better reflect CNS-associated pathological alterations than peripheral blood transcriptomic changes.

**Figure 4:**
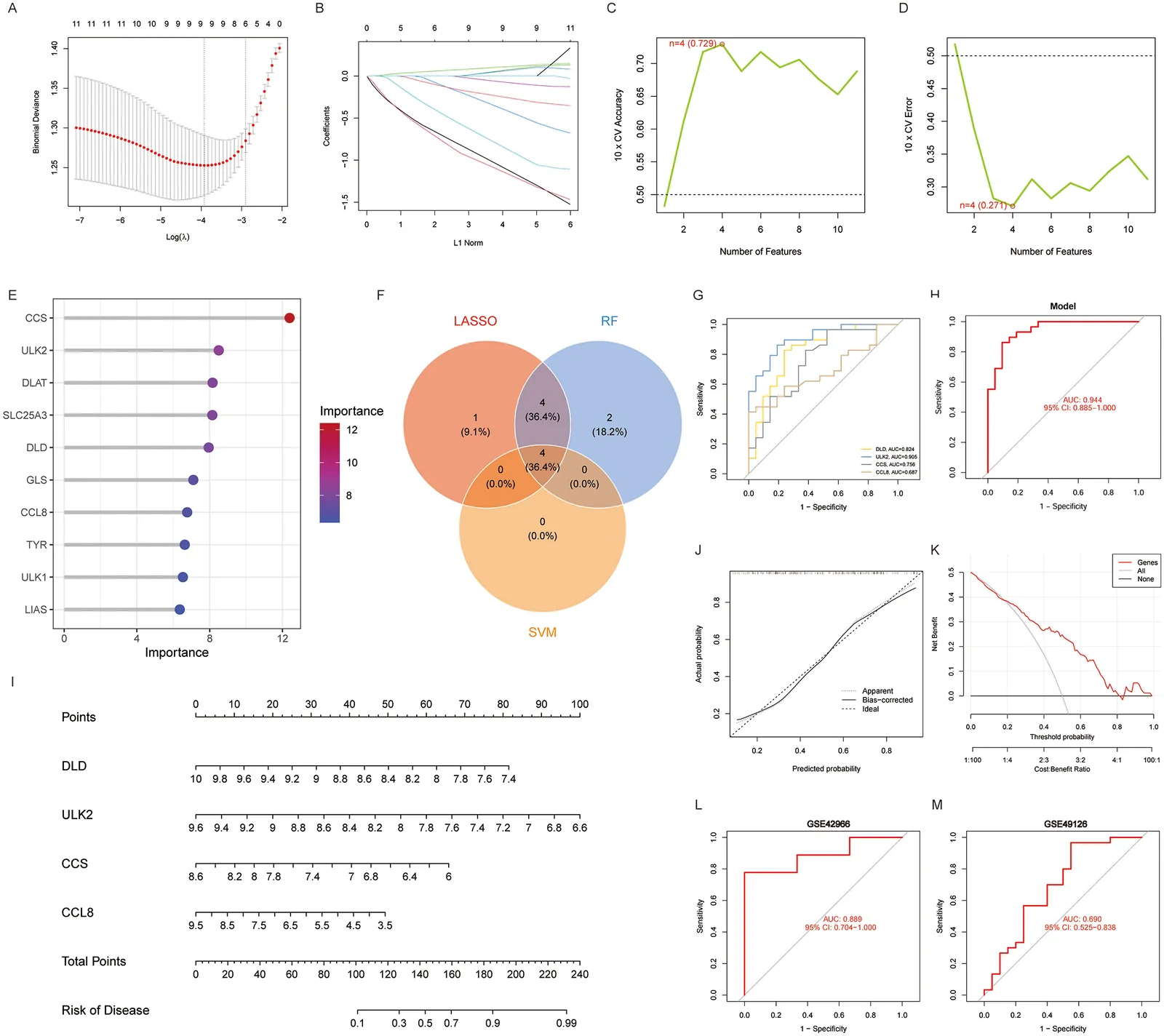
Screening of DECuAGs-related hub genes and establishment of a PD risk model. **(A)** Cross-validation in LASSO regression for selecting the optimal penalty parameter (λ). Error bars represent the standard error of the mean deviance at each λ. **(B)** DECuAGs profiles with LASSO coefficients. Each curve represents the trajectory of a coefficient’s value against the L1 Norm as λ increases. Determination of the optimal number of features for the SVM model using 10-fold cross-validation, where prediction accuracy **(C)** and prediction error **(D)** were shown. **(E)** Top 10 variable importance rankings in the RF model. **(F)** Venn plot depicting 4 intersecting genes selected by three machine-learning models. **(G)** ROC analysis for evaluating the diagnostic potential of 4 hub genes in the test cohort. **(H)** ROC analysis for evaluating the diagnostic potential of the integrated predictive model in the test cohort. **(I)** Predictive nomogram for PD risk based on 4 hub genes. **(J)** Calibration curve of the predictive model. **(K)** Clinical decision curve analysis for the predictive model. **(L)** ROC analysis for the separate dataset GSE42966 (brain). **(M)** ROC analysis for the separate dataset GSE49126 (blood).

### Hub DECuAGs characteristics and PD subclusters

We further elucidated the possible effects of the four DECuAGs in Parkinson’s disease. The expressions of all these genes were significantly downregulated in patients with PD (**Figure 5A**). Their chromosomal locations were shown in **Figure 5B**, in which *ULK2* and *CCL8* were found adjacent. Meanwhile, we performed a correlation analysis for the hub DECuAGs and found that *ULK2* was significantly positively correlated with *CCL8* (**Figure 5C**). Further analysis using GSEA explored the association of hub DECuAGs expression with multiple biological functions and pathways in PD (|NES| > 1, adjusted P value < 0.05; **Supplementary Table S8, 9**). The 10 most significant GO and KEGG pathways of each hub gene were displayed in **Supplementary Figures S3A–G**, many of which were related to the immune and nervous systems. In GO analyses, *CCL8* expression indicated positive correlation with “GOBP_IMMUNE_EFFECTOR_PROCESS”, “GOBP_LEUKOCYTE_PROLIFERATION”, and “GOBP_REGULATION_OF_LEUKOCYTE_PROLIFERATION”. *DLD* expression showed positive correlations with “GOCC_PRESYNAPSE” and “GOCC_SYNAPTIC_MEMBRANE”. And the expression of *ULK2* was negatively related to synapses, such as “GOBP_REGULATION_OF_POSTSYNAPTIC_MEMBRANE_POTENTIAL”. The KEGG terminology suggested that all *ULK2, DLD*, and *CCL8* expression was closely related to “KEGG_CYTOKINE_CYTOKINE_RECEPTOR_INTERACTION”. Both *ULK2* and *CCL8* expression were strongly associated with “KEGG_INTESTINAL_IMMUNE_NETWORK_FOR_IGA_PRODUCTION”. ULK2 expression was also correlated with “KEGG_NEUROACTIVE_LIGAND_RECEPTOR_INTERACTION”. *DLD* expression was associated with “KEGG_AXON_GUIDANCE” and “KEGG_CELL_ADHESION_MOLECULES_CAMS”. While the expression of CCL8 was correlated with “KEGG_NEUROTROPHIN_SIGNALING_PATHWAY”. Additionally, we input the normalized merged gene expression matrix and selected 50 HALLMARK gene sets from the Molecular Signature Database (MsigDB, v.7.5.1) as the reference set for conducting ssGSEA. Concurrently, we performed correlation analyses between the HALLMARK pathway and the hub DECuAGs. As shown in **Supplementary Figure S3H**, we observed that numerous classical pathways associated with inflammatory responses were significantly linked to these four genes, particularly *DLD*, such as “HALLMARK_INTERFERON_GAMMA_RESPONSE”, “HALLMARK_INTERFERON_ALPHA_RESPONSE”, “HALLMARK_INFLAMMATORY_RESPONSE”,

**Figure 5:**
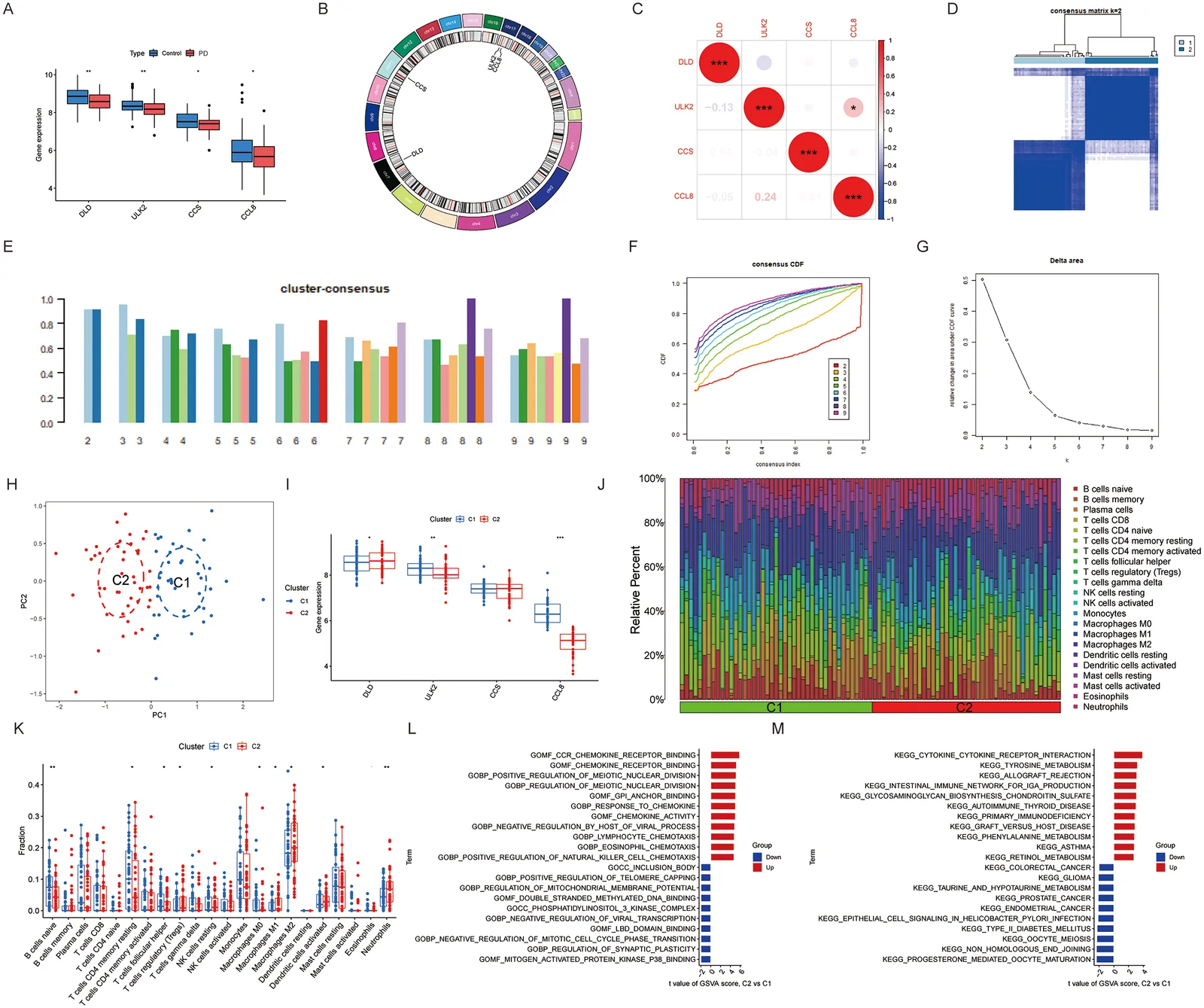
Comprehensive analysis of DECuAGs-related hub genes and their consensus clustering analysis. **(A)** Box plot comparing the mRNA expression levels of 4 hub DECuAGs between PD and the controls. **(B)** Circos plot of the genomic landscape for 4 hub DECuAGs. **(C)** Correlation heatmap of 4 hub DECuAGs. **(D)** Consensus matrix heatmap for k = 2. **(E)** Cluster-consensus plot for k = 2-9. **(F)** Consensus cumulative distribution function curves for k = 2-9. **(G)** Delta area plot for k = 2-9. **(H)** Principal component analysis for validating the robustness of the two clusters. **(I)** Box plot comparing the mRNA expression levels of 4 hub DECuAGs in the two clusters. **(J)** Landscape of 22 immune cell infiltration in the two molecular subtypes. **(K)** Box plot displaying estimated fractions of 22 immune cell types in each cluster. **(L)** Differential GO term activity between the two clusters assessed by GSVA. **(M)** Differential KEGG pathways activity between the two clusters assessed by GSVA. *P < 0.05, **P < 0.01, and ***P < 0.001.

“HALLMARK_IL6_JAK_STAT3_SIGNALING”, “HALLMARK_IL2_STAT5_SIGNALING”, and “HALLMARK_COMPLEMENT”.

In addition, we performed consensus clustering on the differential expression of these genes and divided 85 PD patients into different groups. The results indicated that the clustering was most stable when k = 2 (**Figures 5D–G**). Therefore, we chose to divide the PD samples across two distinct clusters, C1 and C2, which can be well distinguished in the PCA plot (**Figure 5H**). In the two clusters, *ULK2* and *CCL8* were found significantly upregulated in C1, while *DLD* was markedly upregulated in C2 (**Figure 5I**). The differences of the infiltrating immune cells in the PD subtypes were revealed in **Figures 5J** and **K**. Multiple distinctions were detected in the immune microenvironment across the two clusters, with increased infiltration of naive B cells, resting CD4 memory T cells, and follicular helper T cells in C1, and significant upregulation of infiltration of all types of macrophages (M0, M1, and M2), neutrophils, regulatory T cells, resting NK cells, and activated dendritic cells in C2. Additionally, we employed GSVA to investigate biofunctional enrichment within PD subtypes. GO terminology suggested that “GOBP_REGULATION_OF_MITOCHONDRIAL_MEMBRANE_POTENTIAL” and “GOBP_REGULATION_OF_SYNAPTIC_PLASTICITY”, which related to neurometabolism, were enriched in C1. The “GOBP_NEGATIVE_REGULATION_BY_HOST_OF_VIRAL_PROCESS”, “GOBP_LYMPHOCYTE_CHEMOTAXIS”, “GOBP_EOSINOPHIL_CHEMOTAXIS”, and “GOBP_POSITIVE_REGULATION_OF_NATURAL_KILLER_CELL_CHEMOTAXIS”, which were related to immunity, were significantly activated in C2 (**Figure 5L**). Similarly, KEGG analysis indicated the terms associated with immune system dysfunction were concentrated in C2, such as “KEGG_ALLOGRAFT_REJECTION”, “KEGG_INTESTINAL_IMMUNE_NETWORK_FOR_IGA_PRODUCTION”, “KEGG_AUTOIMMUNE_THYROID_DISEASE”, “KEGG_PRIMARY_IMMUNODEFICIENCY”, “KEGG_GRAFT_VERSUS_HOST_DISEASE”, and “KEGG_ASTHMA”. Meanwhile, the “KEGG_TYPE_II_DIABETES_MELLITUS” was primarily increased in C1, indicating an association with metabolic disturbance (**Figure 5M**). Briefly, C1 was metabolic, while C2 was immune-related. According to these results, we inferred that the DECuAGs existing in the Parkinson’s disease nervous system were correlated with energy metabolism and immune function.

### Hub DECuAGs correlated with immune cell infiltration in PD

To further delve into the immune infiltration in PD and its relationship with hub DECuAGs, we performed CIBERSORT analyses on the merged dataset. The comparison analysis of immune-related features between the PD and control groups was depicted in **Supplementary Figure S4A**. The boxplot of the CIBERSORT result showed higher ratios of resting NK cells, M0 macrophages, M1 macrophages, and activated dendritic cells in PD patients. However, the ratios of plasma cells, resting memory CD4 T cells, activated memory CD4 T cells, and eosinophils were reduced in comparison with controls (**Supplementary Figure S4B**). In addition, **Supplementary Figure S4C** revealed the interactions between the immune cells. We further performed Spearman analyses between hub DECuAGs and various types of immunocytes within the PD group (**Supplementary Figure S4D**). Results showed that *ULK2* was positively related to activated dendritic cells, eosinophils, and resting mast cells, while it was negatively associated with M0 macrophages, especially with M1 macrophages. *DLD* exhibited a positive correlation with activated NK cells. *CCS* was positively correlated with CD8 T cells but negatively correlated with follicular helper T cells. *CCL8* was markedly positively associated with neutrophils and negatively associated with naïve CD4 T cells. The correlations of ULK2 expression with the abundance of macrophages in the PD group were displayed in **Supplementary Figure S4E**. ULK2 expression showed a moderate negative correlation with M0 macrophages (R = -0.35, P<0.05) and a significantly strong negative correlation with M1 macrophages (R = -0.57, P<0.0005). Comprehensively, the correlation network heatmap shown in **Supplementary Figure S4F** displayed strong associations of the expression of hub DECuAGs with the immune cell infiltration level, while also revealing the close interactions among immune cells in PD.

### Analysis of publicly available scRNA-seq data from the human substantia nigra

To investigate cell composition changes associated with PD, we analyzed scRNA-seq data of the substantia nigra pars compacta (SNpc). After the quality control process (**Supplementary Figures S5A, B**), a total of 144977 cells were retained for normalization, including 63721 cells from the control group and 81256 cells with PD. We then identified 3000 genes with the highest variation coefficients for the subsequent in-depth research (**Supplementary Figure S5C**). The visualization of the data after dimensionality reduction indicated that each sample was uniformly distributed (**Figure 6A**). Based on the quality assessment of PCA, we determined 20 to be the optimal number of principal components (**Supplementary Figures S5D, E**) and presented the characteristic genes of the main PCA modules (**Supplementary Figures S5F, G**). All cells were categorized into 8 clusters after clustering analysis and visualized in the UMAP plot (**Figure 6B**). The different expressed marker genes in each cluster were then identified (**Supplementary Figure S5H**; **Supplementary Table S10**). According to these results, we manually annotated seven main cell types, including two immune cell species: T cells expressing the typical marker genes *CD2* and *THEMIS*, and microglia (MG) expressing *CD74* and *ITGAM*. The remaining five types of non-immune cells included astrocytes (AST) with typical marker genes *SLC1A2*, *AQP4*, *GFAP*, and *ALDH1L1*; endothelial cells (ENT) with *FLT1*, *RGS5*, and *CLDN5*; neurons (NEU) with *MAP2*, *STMN2*, *SYT1*, and *RBFOX1*; oligodendrocytes (ODC) with *PLP1*, *MBP*, *MOG*, and *MOBP*; and oligodendrocyte precursor cells (OPC) with *PDGFRA*, *VCAN*, and *MEGF11*. In addition, we found that one cluster of neurons highly expressed *TH*, indicating that they were dopaminergic neurons (DaN). Another cluster of neurons was rich in *RBFOX1*, which we annotated as non-dopaminergic neurons (nDaN) (**Figures 6C–E**). Moreover, we compared the ratios for all cytotypes across the control group and the PD group, laying the groundwork for subsequent exploration of expression patterns related to cuproptosis (**Figure 6F**). To ensure scientific comparability, we randomly downsampled the data to approximately 5,000 cells each and presented the results in a UMAP density plot (**Figure 6G**). Compared with the control group, the proportions of DaNs and nDaNs were significantly reduced in the PD group (all P < 0.001), while the proportions of MGs (P < 0.05), ASTs (P < 0.05), T cells (P < 0.05), ENTs (P < 0.001), OPCs (P < 0.001), and ODCs (P < 0.001) were markedly increased (**Supplementary Table S11**).

**Figure 6:**
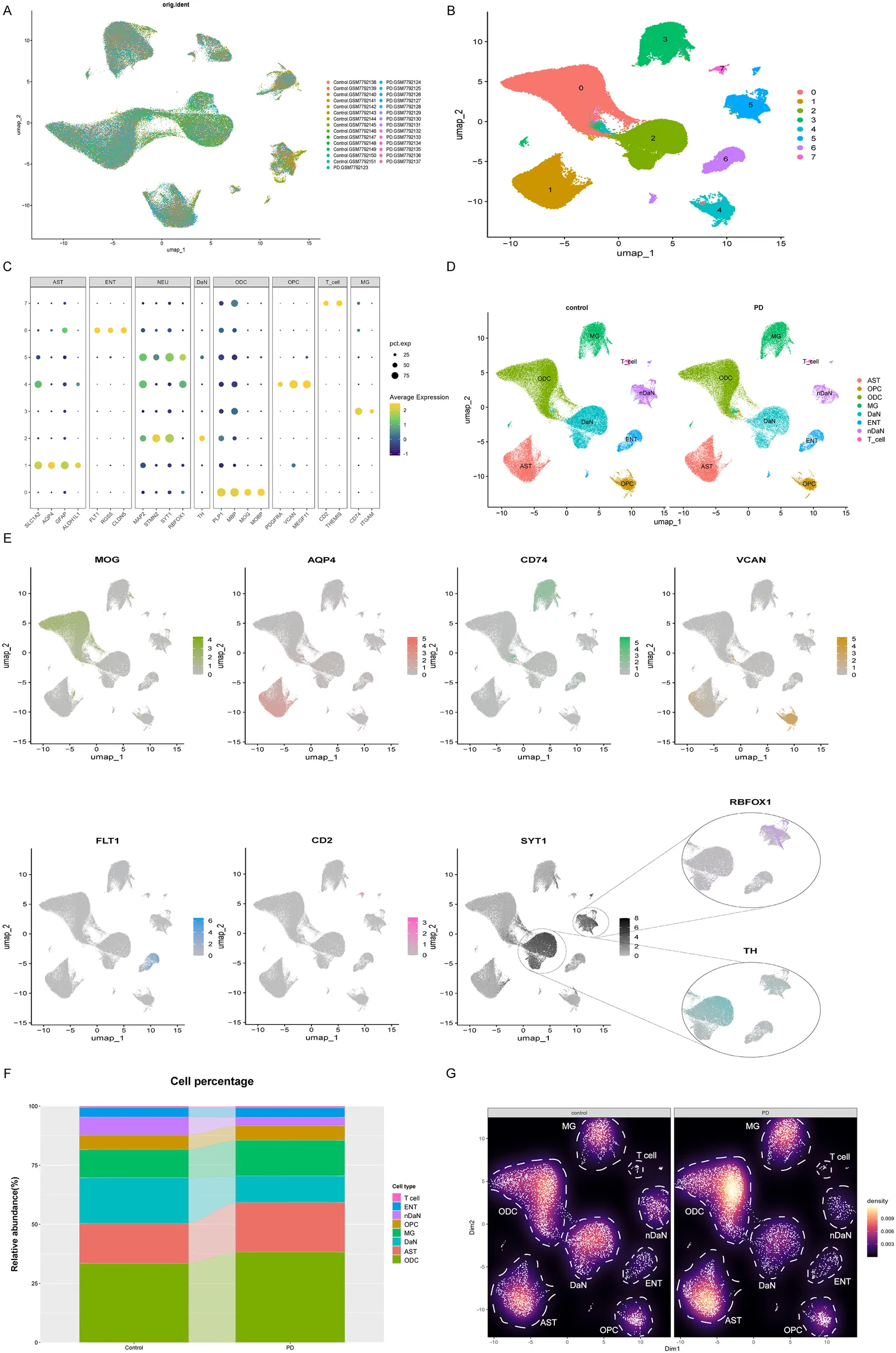
Single-cell RNA sequence analysis of the human substantia nigra public dataset. **(A)** UMAP visualization of the integrated single-cell RNA sequencing (scRNA-seq) dataset, colored by sample origin. **(B)** UMAP visualization of 8 cell clusters. **(C)** Dot plot of marker gene expression for cell type identification. Dot color and size represent the scaled average expression and the percentage of expressing cells, respectively. **(D)** UMAP visualization of annotated cell types in PD and control samples. **(E)** UMAP plots demonstrating typical cell markers defining ODC (MOG), AST (AQP4), MG (CD74), OPC (VCAN), ENT (FLT1), T cell (CD2), and NEU (SYT1). NEU is subdivided into DaN (TH) and nDaN (RBFOX1). **(F)** Comparative analysis of cell type abundance between PD and the controls. **(G)** UMAP density plot of PD samples versus control samples. Each point represents an individual cell.

### Single-cell characterization of hub genes linked to cuproptosis in PD

To elucidate cuproptosis activity at the single-cell level in PD, we first depicted highly heterogeneous landscapes of cuproptosis-associated gene co-expression within distinct cellular populations in both PD and control groups (**Figures 7A, B**). We calculated the average CuAGs expression levels as indicators of cuproptosis activity and made them comparable. Compared to the control group, the PD group exhibited significantly elevated cuproptosis activity, particularly evident in ASTs, DaNs, and MGs (**Figure 7C**; **S5I**). The differentially expressed genes were then compared across various cell types between the PD and control groups. Unsurprisingly, we observed that the cuproptosis marker gene *FDX1* was upregulated in most cell types within the PD group, including DaNs, nDaNs, ASTs, MGs, and ODCs, consistent with previous findings. Concurrently, we discovered that the four previously identified cuproptosis-associated hub genes were differentially expressed primarily within ASTs, DaNs, and MGs in the PD group. Compared to the control group, *CCS* and *DLD* decreased significantly in ASTs in PD. Additionally, *ULK2* and *CCL8* showed markedly reduced expression in MGs, while *DLD* and *CCS* exhibited remarkably diminished expression in DaNs (**Figure 7D**; **Supplementary Figures S5J, K**). UMAP analysis further visualized cell-type-specific expression patterns of these genes, comparing PD and control groups (**Figure 7E**). Combined with the violin plots shown in **Supplementary Figure S5L**, it was not difficult to conclude that *CCS* was expressed predominantly in ASTs, *ULK2* and *CCL8* primarily in MGs, and *DLD* mainly in DaNs, with all these genes showing considerably reduced levels of expression in the PD group.

**Figure 7:**
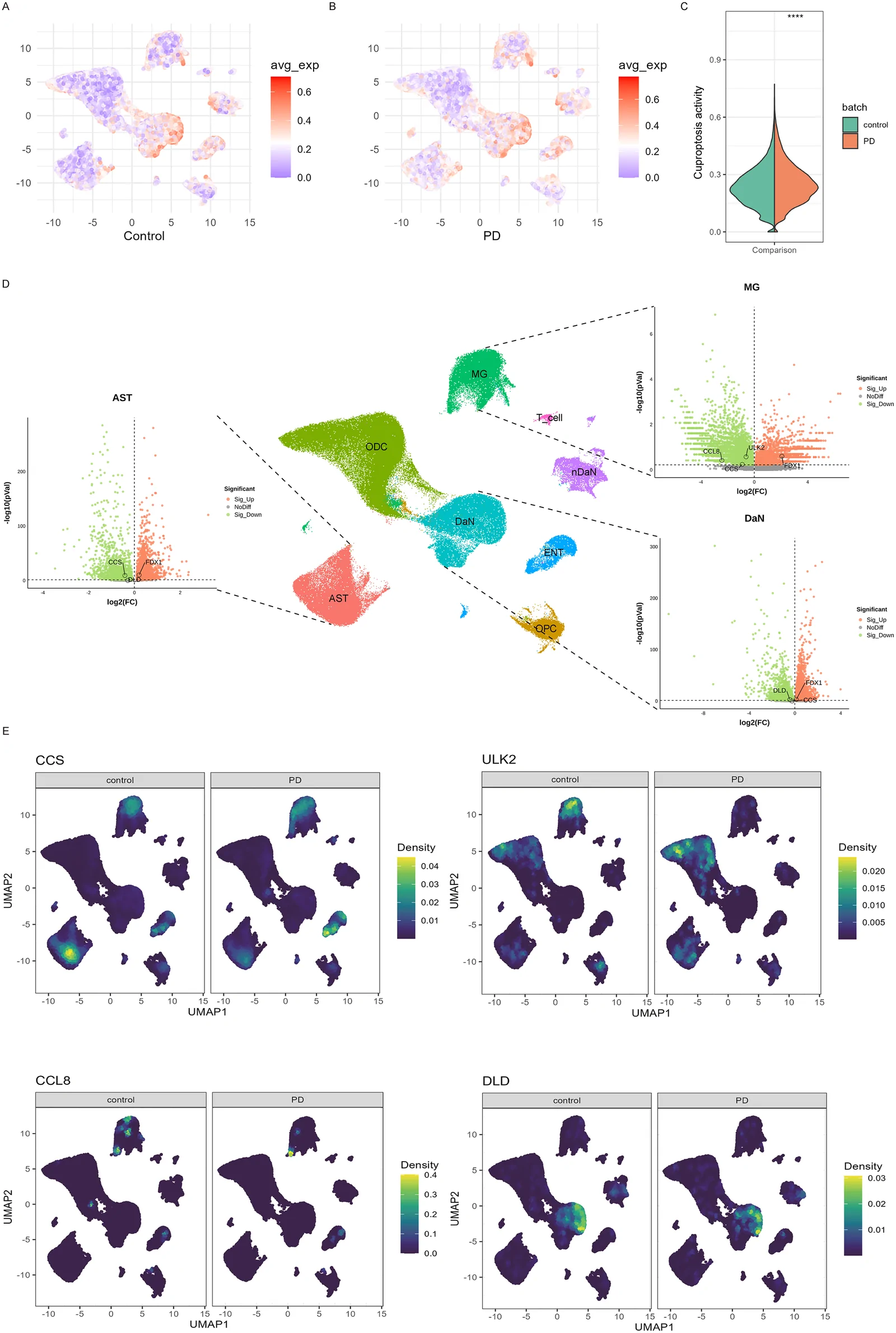
Analysis of cuproptosis activity and its associated hub genes in PD and controls. **(A, B)** Single-cell transcriptomic landscape projected by cuproptosis-associated gene expression in the Control group and the PD group. **(C)** Violin plot comparing cuproptosis activity between PD and the controls. ****P < 0.0001. **(D)** Composite volcano plots demonstrating hub DECuAGs and *FDX1* within astrocytes, microglia, and dopaminergic neurons between PD and the controls. **(E)** Distribution and expression profiles of hub DECuAGs: *CCS*, *ULK2*, *CCL8*, and *DLD*.

### Cell-type-specific in silico perturbation analysis of hub DECuAGs

To further explore the potential functional significance of the cell-type-specific expression patterns of the four hub DECuAGs, we performed cell-type-specific in silico perturbation analyses using virtual gene knockout and overexpression in their predominant cell populations. Simulated perturbation of *DLD* in DaNs predominantly affected neuronal and synaptic processes, including neurotransmitter transport and synaptic vesicle organization (**Supplementary Figure S6**). In ASTs, perturbation of *CCS* mainly influenced protein folding, proteostasis, and endoplasmic reticulum-associated pathways, particularly under PD conditions (**Supplementary Figure S7**). In MGs, perturbation of *ULK2* preferentially altered inflammatory and immune-related signaling pathways, including NF-κB, Toll-like receptor, TNF, and MAPK signaling (**Supplementary Figure S8**), whereas perturbation of *CCL8* was associated with biological processes involved in immune regulation, cell communication, and synaptic organization (**Supplementary Figure S9**). Moreover, the predicted perturbation-associated functional profiles differed between control and PD conditions, suggesting disease-associated remodeling of the underlying gene regulatory networks. Overall, these in silico perturbation analyses provide hypothesis-generating computational support for potential cell-type-specific functional roles of the four hub DECuAGs and highlight the biological relevance of their preferential expression patterns in dopaminergic neurons, astrocytes, and microglia.

### Cell-cell communication alterations

We further explored alterations in intercellular communication involved in the pathogenesis of PD, based on gene expression information obtained from the scRNA-seq analysis above using the CellChat online ligand-receptor databases. First, we compared the aggregated cell communication networks of the PD and the control group, revealing significant differences between them, especially between MGs and other cells, both in terms of quantity and weight (**Supplementary Figure S10A**). Subsequently, we focused on the interactions between the hub DECuAGs-related cell subpopulations (MGs, ASTs, and DaNs), revealing that MG-AST interactions were significantly reduced in the PD group compared to controls, while MG self-interactions were absent in the PD group. In addition, we observed that in the control group, DaNs sent weak signals to MGs, whereas in the PD group, DaNs did not send any signals to MGs (**Figure 8A**). Combined analysis of the interaction strength network and heatmap among MGs, ASTs, and DaNs also showed that the signals emitted by MGs in the PD group were significantly reduced versus the control group (**Figure 8B**).

**Figure 8:**
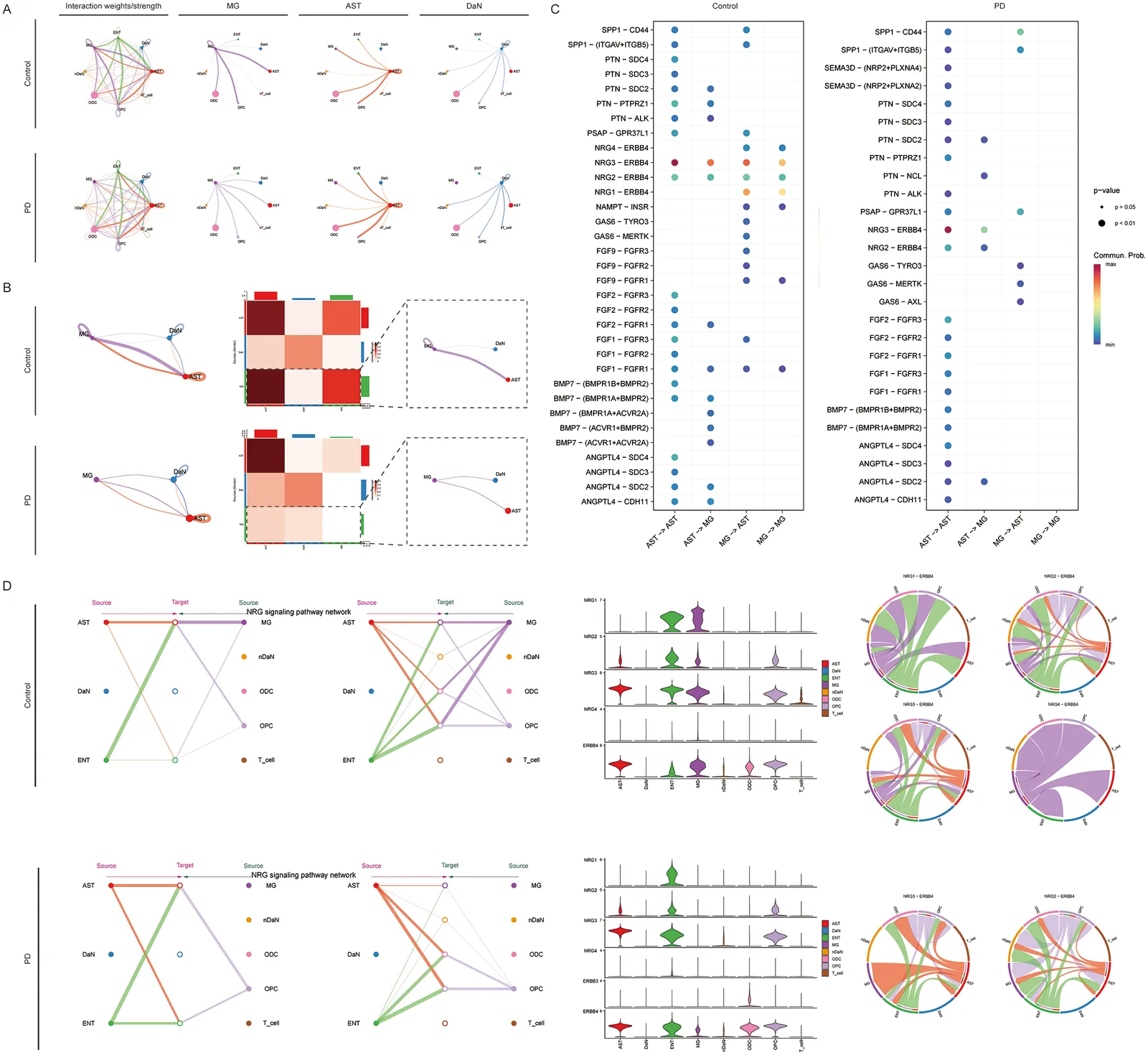
Intercellular communication in PD and the controls. **(A)** Circle plots illustrating the network of interaction strengths among the 8 major cell populations in PD and the controls. The thickness of the edges indicates the strength of the interactions, while the color denotes the identity of the source cell population. **(B)** Circle plots and heatmaps revealing the interaction strength across microglia and astrocytes in PD and the controls. Color gradient reflects the scaled interaction strengths. **(C)** Bubble plot highlighting ligand-receptor interactions across microglia and astrocytes in PD compared to controls. Dot size indicates statistical significance, while color denotes communication probability. **(D)** Integrated visualization of the NRG signaling pathway in PD and controls. Hierarchical plots show the intercellular communication network of the NRG signaling pathway (left). Violin plots display the expression distributions of the same six NRG-pathway genes—NRG1, NRG2, NRG3, NRG4, ERBB3, and ERBB4—across the major cell types in both the Control and PD groups (middle). Chord plots show cellular communication at the ligand-receptor pair level within the NRG signaling pathway (right). Identical colors denote identical cells.

In our in-depth analysis, we calculated the communication probability between ligand-receptor pairs to discover and reveal the signaling pathways that change during the progression of Parkinson’s disease. We found 392 important ligand-receptor pairs within the control group and 482 pairs within the PD group. These ligand-receptor pairs were involved in 22 identical signaling pathways, including neuregulin (NRG), growth arrest-specific (GAS), and VISFATIN, which were related to metabolism. It also contained WNT-related integration sites (WNT), non-classical WNT signaling pathways (ncWNT), transforming growth factor-β (TGFβ), and other immune-related signaling pathways. In addition, we observed that interleukin-16 (IL-16) signals were transmitted from T cells to DaNs exclusively in the PD group. At the same time, insulin-like growth factor (IGF) pathways were identified solely in ENTs within the control group (**Supplementary Tables S12, 13**). Notably, we observed that the NRG pathway was the strongest intracellular signal in both the PD group and the controls, especially from MGs, ASTs, ENTs, and OPCs (**Supplementary Figure S10B**). However, NRG pathway signaling from MGs was dramatically reduced in the PD group, suggesting that the altered NRG pathway in PD may be responsible for the attenuated MG-related cellular interactions demonstrated in **Figure 8B**; **Supplementary Figures S10A, C**). Since we were concerned with identifying high-probability signaling pathways in cell populations associated with hub DECuAGs, we narrowed our study to MGs and ASTs. Coincidentally, we noticed that cellular communication in MGs and ASTs in controls may be predominantly through the NRG signaling pathway, whereas PD was markedly diminished (**Figure 8C**). These results create a possible inference that the remarkably altered NRG signaling pathway in the cuproptosis-marked cell populations may be associated with the progression of PD.

We then conducted a detailed analysis of the NRG signaling pathway (**Figure 8D**). In the control group, MG was a major sender and receiver in the NRG pathway, but not in PD (**Figure 8D**, left panels, **Supplementary Figure S10D**). In parallel, we also noted that ligands of this pathway were barely expressed within MGs in the PD group (**Figure 8D**, middle panels). Subsequently, we computed Euclidean distance scores of every ligand-receptor pair and found that NRG3-ERBB4 was the predominant component in both the PD group and the controls. NRG1-ERBB4 exhibited a greater distance in controls, whereas NRG2-ERBB4 was more distant in PD (**Supplementary Figure S10E**). The cellular interactions involved in MGs and ASTs of the NRG pathway were shown as string diagrams (**Figure 8D**, right panels). In controls, the cellular interactions between MGs and ASTs in the NRG pathway were NRG1-ERBB4, NRG2-ERBB4, NRG3-ERBB4, and NRG4-ERBB4. Together, NRG2-ERBB4 and NRG3-ERBB4 constituted the intrinsic NRG signaling pathway of AST-AST interactions. As for MG-MG interactions, NRG1-ERBB4, NRG2-ERBB4, NRG3-ERBB4, and NRG4-ERBB4 simultaneously constituted the NRG signaling pathway. In the PD group, we observed that ASTs and MGs communicate in the NRG pathway only through NRG2-ERBB4 and NRG3-ERBB4, while ASTs themselves communicated in the same way as in the control group. And the NRG signaling pathway was lost in MG-MG interactions.

### Expression analysis of DECuAGs in the A53T α-synuclein mouse model of PD

The A53T α-synuclein mouse model is a widely used model that reproduces the key protein pathology—progressive aggregation of α-synuclein—in Parkinson’s disease (30). We successfully established this model through stereotaxic injection of AAV9-A53T virus into the substantia nigra region of mice, subsequently employing them for follow-up validation experiments (**Figure 9**). To further validate the co-expression patterns and expression levels of the 4 hub genes, we performed mIF and Western blot analyses using these injected mice. We detected a marked increase in FDX1 fluorescence intensity in the mouse brain on the PD lesion side, suggesting increased cuproptosis-related molecular activity accompanying dopaminergic fiber loss in PD (**Figure 10A**). Furthermore, by examining the colocalization of FDX1 with TH, IBA1, and GFAP in the SN of PD and control groups, we observed increased FDX1-related signals in dopaminergic neurons, microglia, and astrocytes within PD (**Figures 10B–E**). Concurrently, we observed significantly reduced co-localization in PD for the hub protein pairs: DLD with TH (**Figure 10B**), ULK2 with IBA1 (**Figure 10C**), CCS with GFAP (**Figure 10D**), and CCL8 with IBA1 (**Figure 10E**). This indicated that in PD, DLD expression decreased in dopaminergic neurons, ULK2 and CCL8 were downregulated in microglia, and CCS levels were reduced in astrocytes. Additionally, results from Western blot analysis revealed that compared to the control group, expression levels of DLD, ULK2, CCS, and CCL8 were significantly reduced in the PD group, while FDX1 was markedly elevated (**Figure 10F**). These findings support an association between dysregulation of the four hub DECuAGs and increased cuproptosis-related molecular alterations in the PD model.

**Figure 9:**
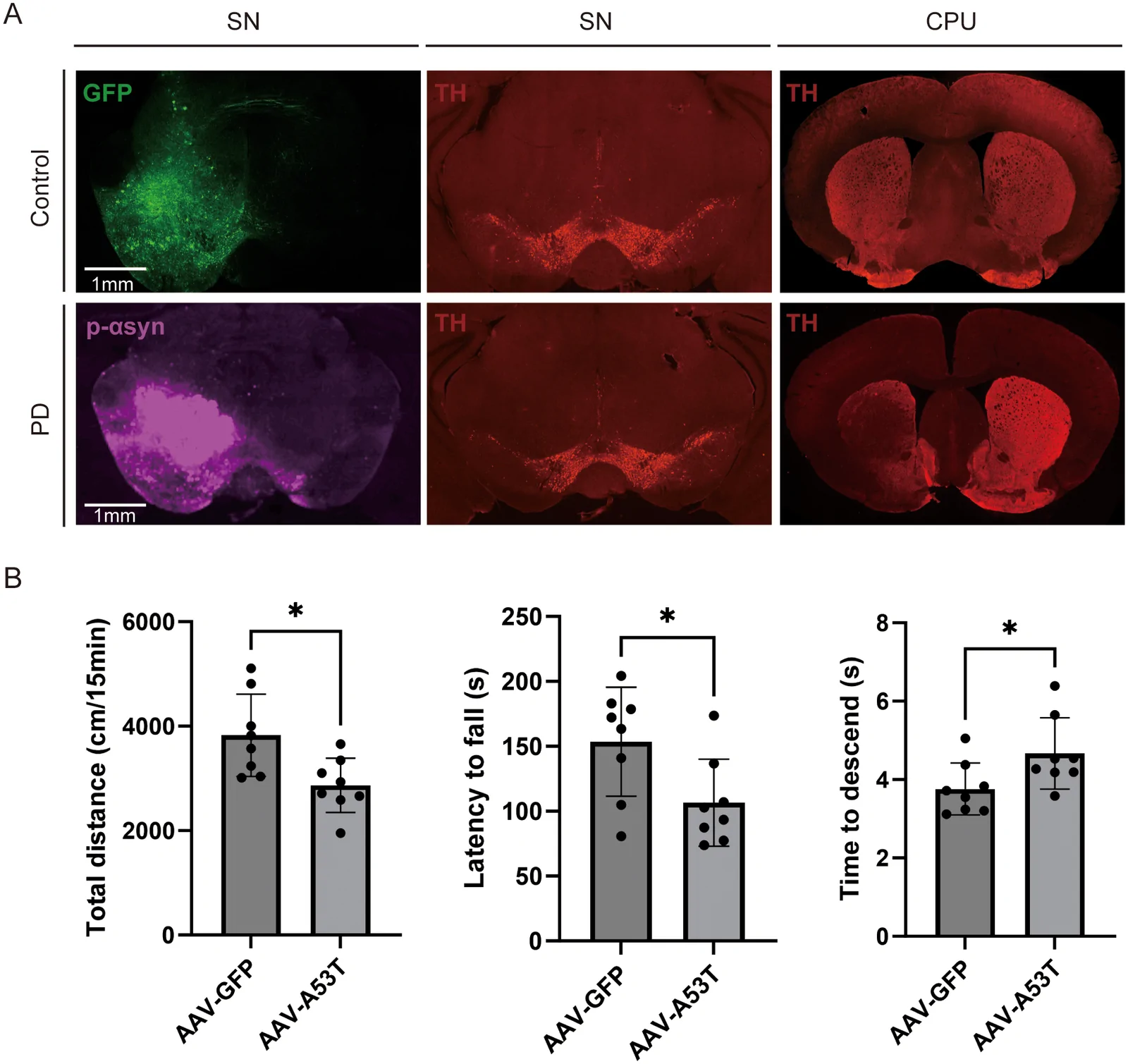
The establishment of the A53T α-synuclein mouse model. **(A)** Immunofluorescent staining of the SN and CPU in mice 12 weeks after AAV9 injection, revealing the significant reduction of dopaminergic neurons and their fibers in the A53T α-synuclein mouse model. Green fluorescence (GFP). Pink fluorescence (p-αsyn). Red fluorescence (TH). Scale bar: 1 mm, n = 1. **(B)** Impaired motor performance in A53T α-synuclein mice compared to controls. Results of the open field test (left panel), the rotarod test (middle panel), and the pole test (right panel). All data are presented as mean ± SD. *P<0.05 vs AAV9-GFP group (Control), n = 8.

**Figure 10:**
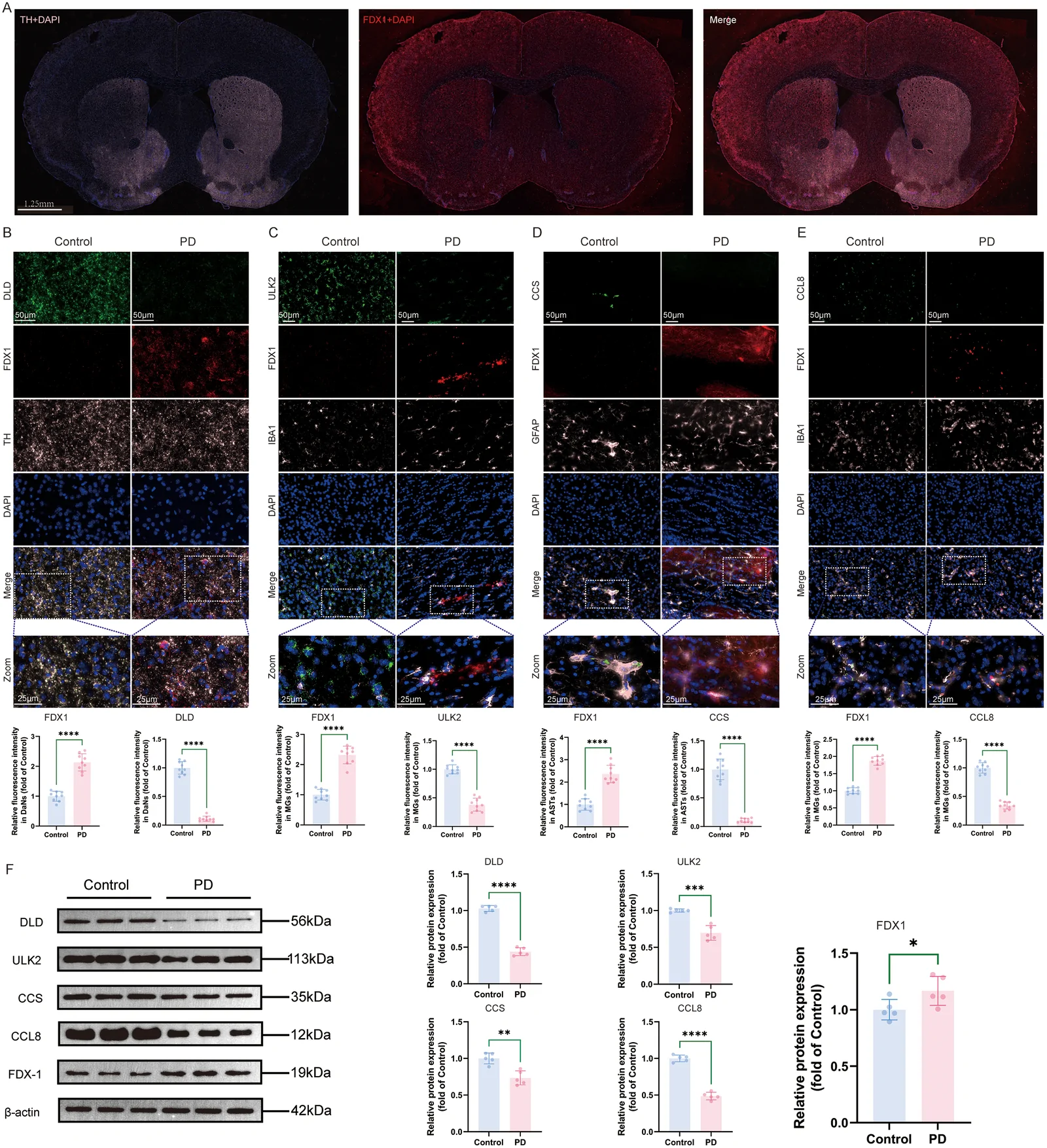
Expression of DLD, ULK2, CCS, CCL8, and FDX1 in dopaminergic neurons, microglia, and astrocytes from A53T α-synuclein mice and the controls. **(A)** Representative mIF images showing co-localization of FDX1 and TH in the CPU of an α-synuclein A53T mouse, indicating increased cuproptosis-related molecular activity accompanying dopaminergic fiber loss in the pathological progression of PD. Red fluorescence (FDX1). Pink fluorescence (TH). Blue fluorescence (DAPI). Scale bar: 1.25 mm. **(B)** Representative mIF images showing co-localization of DLD, FDX1, and TH, and their relative fluorescence intensity. **(C)** Representative mIF images showing co-localization of ULK2, FDX1, and IBA1, and their relative fluorescence intensity. **(D)** Representative mIF images showing co-localization of CCS, FDX1, and GFAP, and their relative fluorescence intensity. **(E)** Representative mIF images showing co-localization of CCL8, FDX1, and IBA1, and their relative fluorescence intensity. n = 3. Relative fluorescence intensity was calculated from three fields per sample after normalization to the corresponding marker-positive cell number. Green fluorescence (DLD/ULK2/CCS/CCL8). Red fluorescence (FDX1). Pink fluorescence (TH/IBA1/GFAP). Blue fluorescence (DAPI). Scale bar: 50 μm for low magnification; 25 μm for high magnification. **(F)** Representative Western blot images and relative protein expression of DLD, ULK2, CCS, CCL8, and FDX1. Protein levels were normalized to β-actin. n = 5 mice per group. All data are presented as mean ± SD. *P < 0.05, **P < 0.01, ***P < 0.001, and ****P < 0.0001 vs AAV9-GFP group (Control).

### Pharmacological modulation of cuproptosis-related molecular activity in PD model mice

To further evaluate whether the identified molecular alterations were responsive to pharmacological modulation of cuproptosis-related molecular activity, we performed pharmacological intervention experiments using the copper chelator TTM and the copper ionophore-based cuproptosis activator Elesclomol-Cu in the AAV9-A53T α-synuclein mouse model. First, we examined whether cuproptosis-related molecular activity, reflected by FDX1 expression, was associated with changes in the dopaminergic neuronal marker TH. As shown in **Figure 11A**, compared with the Control + Vehicle group, the PD + Vehicle group exhibited increased FDX1 expression, accompanied by a marked reduction in TH expression. Importantly, FDX1 expression showed bidirectional responsiveness to pharmacological modulation, with TTM reducing FDX1 expression and Elesclomol-Cu further increasing FDX1 expression in PD mice. In parallel, TTM treatment partially restored TH expression, whereas Elesclomol-Cu aggravated the reduction of TH. These opposite changes suggest a potential association between increased cuproptosis-related molecular activity and reduced dopaminergic neuronal marker expression in the PD model. We next examined whether the four hub DECuAG proteins were responsive to pharmacological modulation of cuproptosis-related molecular activity. Compared with the Control + Vehicle group, the PD + Vehicle group showed decreased protein levels of DLD, ULK2, CCS, and CCL8 (**Figure 11B**). In PD mice, TTM treatment generally increased the expression of these hub DECuAG proteins, whereas Elesclomol-Cu treatment further reduced their expression to varying degrees. Among them, DLD, ULK2, and CCS showed clearer restorative responses to TTM, while CCL8 showed a similar upward trend but with a relatively weaker response. Conversely, Elesclomol-Cu consistently promoted further reductions in the hub DECuAG proteins in the PD background. Together, these findings suggest that FDX1 expression, dopaminergic neuronal marker expression, and hub DECuAG protein alterations were responsive to pharmacological modulation of cuproptosis-related molecular activity in the PD model.

**Figure 11:**
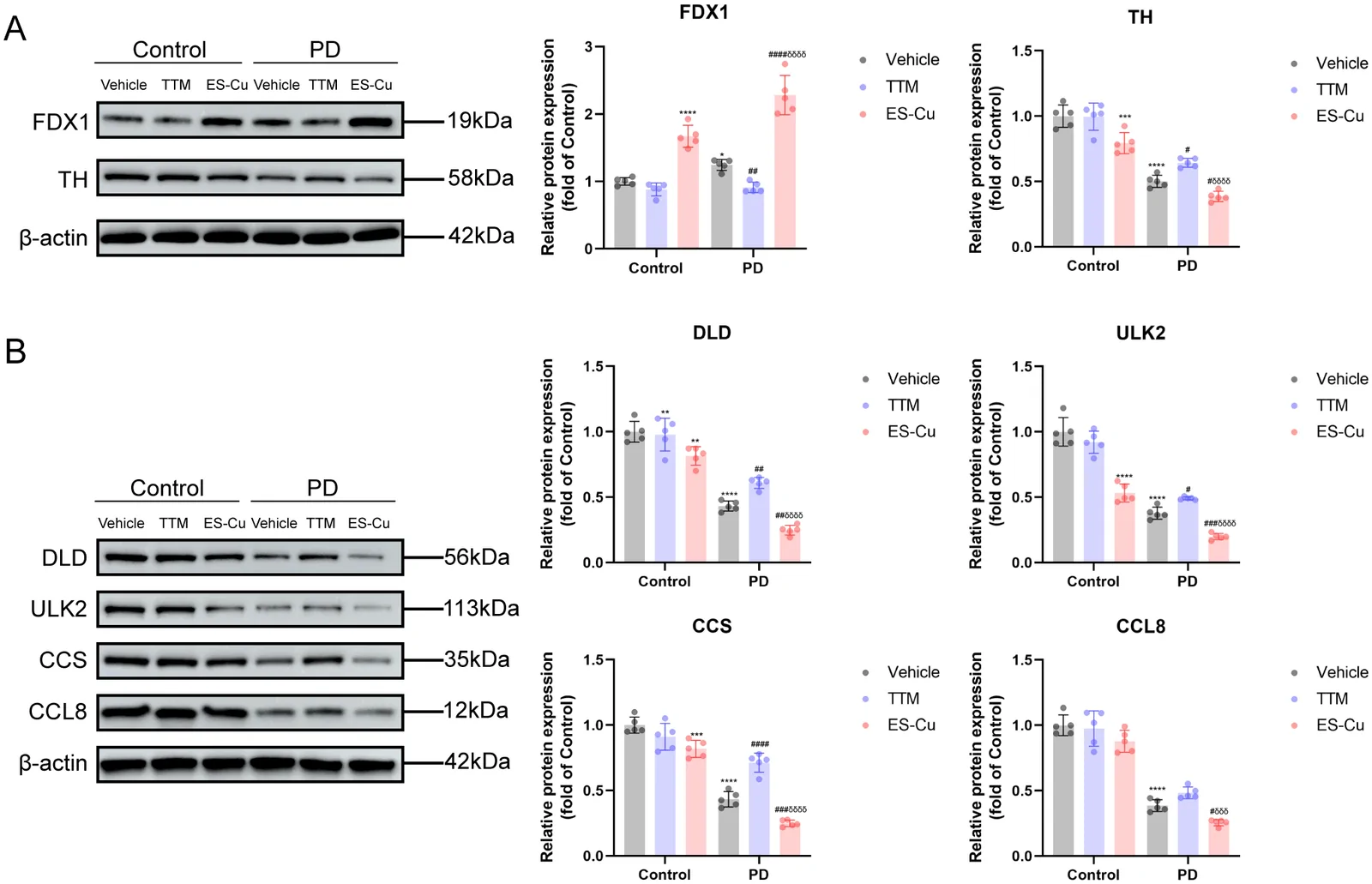
Pharmacological modulation of cuproptosis-related molecular activity in the AAV9-A53T α-synuclein mouse model. **(A)** Western blotting of FDX1 and TH, and quantification of their relative protein expression in substantia nigra tissues from Control + Vehicle, Control + TTM, Control + Elesclomol-Cu, PD + Vehicle, PD + TTM, and PD + Elesclomol-Cu mice. FDX1 was assessed as a responsive marker of cuproptosis-related molecular activity, and TH was used as a dopaminergic neuronal marker. **(B)** Western blotting of DLD, ULK2, CCS, and CCL8, and quantification of their relative protein expression in substantia nigra tissues from the same treatment groups. Protein levels were normalized to β-actin and expressed relative to the Control + Vehicle group. n = 5. All data are presented as mean ± SD. Statistical analysis was performed using two-way ANOVA followed by Šídák’s multiple-comparisons test for pre-specified pairwise comparisons. *P < 0.05, **P < 0.01, ***P < 0.001, ****P < 0.0001 vs Control + Vehicle; ^#^P < 0.05, ^##^P < 0.01, ^###^P < 0.001, ^####^P < 0.0001 vs PD + Vehicle; ^δ^P < 0.05, ^δδ^P < 0.01, ^δδδ^P < 0.001, ^δδδδ^P < 0.0001 vs PD + TTM.

### In silico prediction of candidate compounds targeting hub DECuAG-related proteins

To explore candidate compounds related to the four hub DECuAGs, we performed DSigDB-based in silico screening. A total of 61 drugs were found, comprising 38 components for DLD, 21 compounds for ULK2, whilst only one molecule each was found for CCS and CCL8, with all drugs possessing corresponding enrichment scores (**Supplementary Table S14**). Higher enrichment scores indicate stronger predicted associations between drug molecules and target gene biological networks. According to this ranking, the highest-scoring molecule for each hub DECuAG was selected as a candidate compound for further computational evaluation, including 5,7-Dimethoxyflavone (for *DLD*), Crenolanib (for *ULK2*), Trichostatin A (for *CCS*), and Testosterone (for *CCL8*). These candidate drugs were further studied for their binding feasibility with their corresponding target proteins through molecular docking technology. We screened the most favorable molecular docking patterns based on the Vina scores (kcal/mol) between receptor proteins and drug molecules. The protein-drug interaction complex with the lowest score was considered to have the strongest binding affinity, whilst values below 5 were generally regarded as stable (31). As shown in **Figure 12A**, we discovered four optimal docking conformations for protein-drug pairs: DLD-5,7-Dimethoxyflavone with a binding affinity of -8.6 kcal/mol, ULK2-Crenolanib with -9.7 kcal/mol, CCS-Trichostatin A with -7.4 kcal/mol, and CCL8-Testosterone with -6.6 kcal/mol. The predicted 2D interaction diagrams suggested favorable binding patterns, particularly exhibiting multivalent binding characteristics: hydrogen bonds, van der Waals forces, hydrophobic interactions, and electrostatic interactions coexist, with the drug molecules precisely embedded into the binding pockets of target proteins. Moreover, within each binding pattern, one or more key amino acid residues were found to form high-energy, directionally specific interactions with the drug, acting as “locks” that anchor the drug in its correct position. In the DLD-5,7-Dimethoxyflavone, the Pi-cation interaction with LYS A:54 and the hydrogen bond network with SER A:53 and GLY A:149 were observed. Within the ULK2-Crenolanib, precise positioning was mediated by a water bridge formed between ASN C:217 and ASP A:92. ARG A:196 formed stable hydrophobic contacts with its alkyl side chain, conferring a significant free energy gain to the overall CCS-Trichostatin A binding process. LYS A:49 and ARG A:47 in the CCL8-Testosterone served as pivotal electrostatic anchor points, providing decisive orientation and stabilizing forces for highly specific binding through the formation of robust salt bridges or electrostatic interactions with ligands.

**Figure 12:**
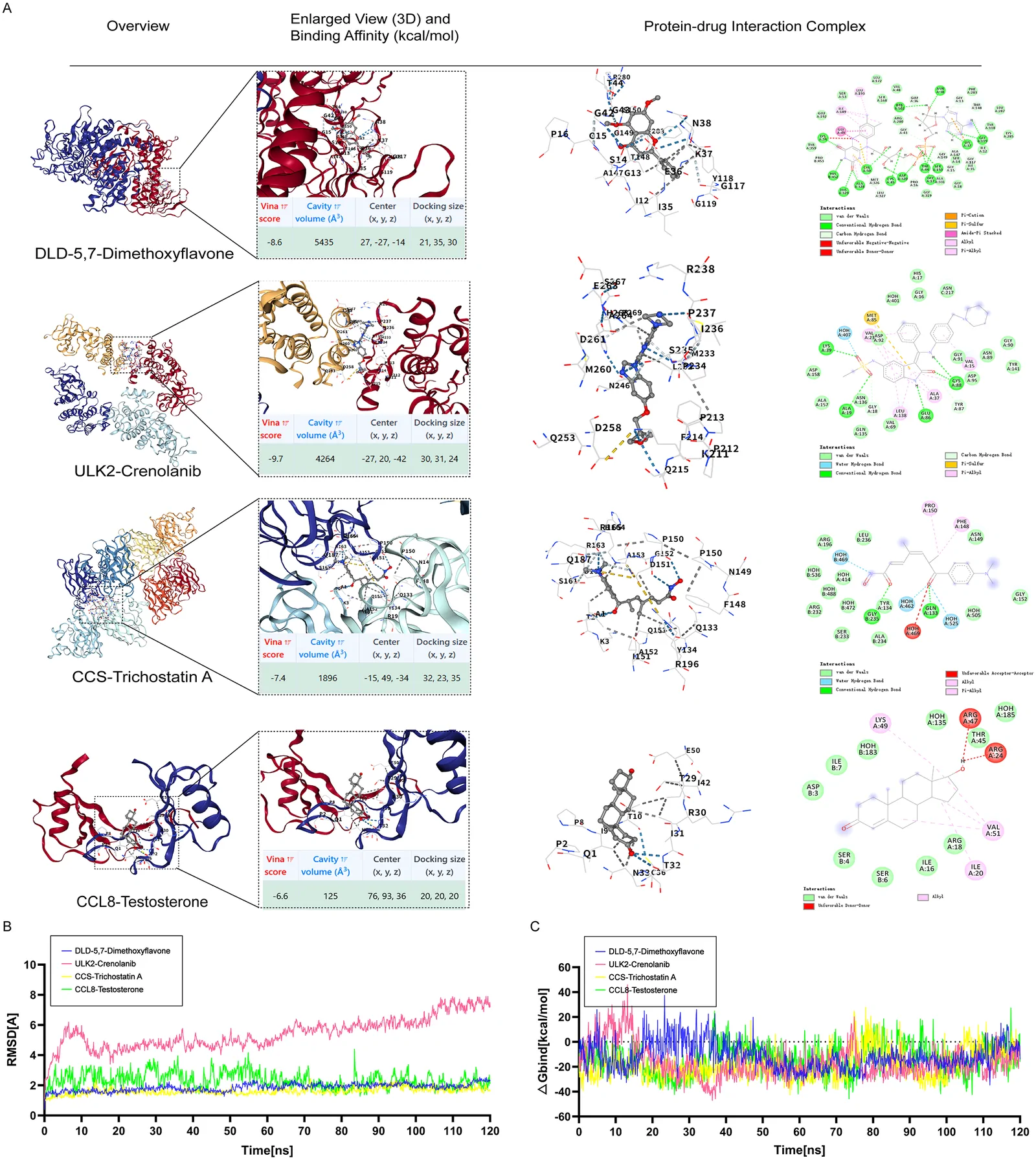
Exploration and validation of potential PD drugs targeting hub DECuAGs. **(A)** Molecular docking results and interaction features between the screened potential drug molecules and the drug target hub DECuAGs proteins. **(B)** RMSD of protein-ligand complexes over molecular dynamics simulation time. The RMSD (in Å) for the backbone atoms of each protein relative to their initial structure is plotted against simulation time (in ns). **(C)** Time-dependent binding free energy (ΔGbind) for the simulated complexes. The free energy of binding (in kcal/mol) is plotted against the simulation time (in ns). In plots **(B, C)**, the blue, red, yellow, and green curves represent DLD-5,7-Dimethoxyflavone, ULK2-Crenolanib, CCS-Trichostatin **(A)**, and CCL8-Testosterone, respectively.

To verify the stability of the four docked complexes and evaluate their dynamic behavior, we performed 120 ns molecular dynamics simulations for each complex. RMSD curves depicted in **Figure 12B** quantify the overall deviation of the docked complexes relative to the initial conformations. The average RMSD values were 1.855 Å (0.405 Å to 2.532 Å), 5.432 Å (0.461 Å to 7.896 Å), 1.618 Å (0.492 Å to 2.445 Å), 2.342 Å (0.398 Å to 4.384 Å), corresponding to DLD-5,7-Dimethoxyflavone, ULK2-Crenolanib, CCS-Trichostatin A, and CCL8-Testosterone, respectively. All four conformational structures fluctuated within 20 ns and subsequently stabilized in the following simulations, indicating dynamic stability under simulated conditions. MM-PBSA binding free energy (ΔGbind) was used to quantitatively predict and compare the strength of intermolecular interactions. The greater the negative value, the stronger the binding force. The average ΔGbind for DLD-5,7-Dimethoxyflavone, ULK2-Crenolanib, CCS-Trichostatin A, and CCL8-Testosterone were: -11.907 kcal/mol, -16.317 kcal/mol, -18.785 kcal/mol, -13.281 kcal/mol, respectively (**Figure 12C**). Overall, these computational results prioritized four candidate compounds targeting hub DECuAG-related proteins for further experimental validation.

## Discussion

This study integrated multi-omics analyses with supportive validation in an AAV9-A53T α-synuclein mouse model and identified *DLD*, *ULK2*, *CCS*, and *CCL8* as cuproptosis-associated hub genes in PD. Our findings revealed cell-type-specific dysregulation of these genes in the PD model and suggested a potential association between cuproptosis-related molecular alterations, metabolic dysfunction, immune abnormalities, and dopaminergic neurodegenerative changes, as further supported by the *in vivo* pharmacological responsiveness of FDX1, hub DECuAG protein levels, and TH expression to TTM and Elesclomol-Cu treatment. Further cell-cell communication analysis suggested altered intercellular signaling, particularly involving the NRG signaling pathway between microglia and astrocytes. Finally, in silico drug–target prediction, molecular docking, and molecular dynamics simulations identified several candidate compounds targeting proteins related to these hub genes. Collectively, these findings provide a multi-omics framework for understanding cuproptosis-associated gene dysregulation in PD and offer candidate biomarkers and therapeutic leads for further validation, while the causal and synergistic roles of these hub genes remain to be determined in future functional studies.

Parkinson’s disease is an advanced neurodegenerative disorder with complex pathogenesis, insidious onset, and a lack of effective long-term treatments. The typical pathophysiology features of PD are α-synuclein protein aggregation and dopaminergic neuron death, although the mechanisms involved need to be further explored. In this study, we joined and integrated three large PD brain tissue RNA-seq datasets from different origins, eliminating batch effects while preserving valuable biological variability to extract clearer, more representative characteristic genes for PD. Through multidimensional, multi-level analyses of the biological functions/pathways of the DEGs, we have confirmed that PD is closely associated with altered mitochondrial metabolism. In addition, we observed significantly upregulated proinflammatory immune infiltration in PD, primarily driven by increased numbers of activated dendritic cells, M0 macrophages, and M1 macrophages. All of these findings were consistent with the recent studies demonstrating that metabolic dysfunction within mitochondria and chronic neuroinflammation, along with their complex interactions, were intimately linked to PD progression (5, 32–34).

The brain contains the second-highest concentration of copper among all organs, with the SN exhibiting the highest copper content, where dopamine neurons are abundant (8). This biologically essential metal is vital for the function of the human nervous system, yet excessive accumulation can prove toxic (35). Available data confirm that cerebral mitochondria are a major candidate target for copper neurotoxicity (36). Moreover, excessive copper can serve as an upstream trigger for neuroinflammation, with this downstream effect primarily mediated by microglia. This copper-induced microglial dysfunction manifests as impaired phagocytic capacity accompanied by enhanced release of proinflammatory cytokines (37, 38). A novel programmed cell death driven by mitochondrial stress and damage—cuproptosis—has also been identified as a unique form of metabolic cell demise (39, 40). Concurrently, it has been closely associated with immunological functions in multiple diseases like Alzheimer’s disease, cerebral cavernous malformation, and hepatocellular carcinoma (41–43). It is noteworthy that existing evidence indicates copper homeostasis abnormalities contribute to the pathogenesis of PD (44). Meanwhile, mounting evidence links cuproptosis to multiple neurodegenerative disorders, including PD, although the precise molecular mechanisms and exact role remain unclear (45, 46). In summary, copper and cuproptosis appear to be closely related to metabolic abnormalities and inflammation induction, and seem to be implicated in the development of PD. As a key focus for our subsequent research, we chose to explore the roles of genes associated with cuproptosis in PD. By intersecting with the CuAGs and the DEGs, we obtained 11 DECuAGs. Four hub DECuAGs: *ULK2*, *DLD*, *CCS*, and *CCL8* were then identified as potential PD biomarkers by machine learning algorithms, and a satisfactory nomogram was created using these four genes to predict PD risk. The lower AUC in the external blood validation dataset also suggests that the generalizability of this hub-gene signature may be tissue dependent. By employing GSEA and ssGSEA analyses, we compared enrichment results at both cohort and single-sample levels for PD and control groups encompassing GO, KEGG, and HALLMARK gene sets. Findings suggested these four hub genes were closely associated with immune regulation within the nervous system, especially *DLD*. Analysis of immune cell infiltration also indicated that all hub DECuAGs correlated with the recruitment of various immune cells. Notably, *ULK2* expression was inversely correlated with the recruitment of M1 macrophages, a type of activated macrophage known to trigger and exacerbate PD by secreting pro-inflammatory cytokines (47). Consequently, we propose that *DLD*, *ULK2*, *CCL8*, and *CCS*, as cuproptosis-associated hub genes identified in this study, may be related to metabolic dysfunction and chronic inflammatory responses during PD progression. Nevertheless, our current data indicate association and co-dysregulation, rather than direct evidence that these genes influence the cuproptosis pathway or constitute a synergistic regulatory network.

According to relevant literature, dihydrolipoamide dehydrogenase (DLD), acting as the E3 subunit for the pyruvate dehydrogenase complex, was one of the earliest cuproptosis-associated genes identified through genome-wide CRISPR-Cas9 screening. However, the precise mechanism of its role in cuproptosis is still uncertain (13). Research has indicated that dihydrolipoamide dehydrogenase deficiency may increase susceptibility to the neurotoxicity of MPTP, malonic acid, and 3-nitropropionic acid in mice, suggesting that DLD may possess neuroprotective properties against neurodegenerative diseases (48). UNC-51-like kinase 2 (ULK2) belongs to the family of serine/threonine kinases, with its most widely recognized function lying in its highly conserved evolutionary significance within the autophagy pathway, including mitophagy—a form of autophagy that targets injured mitochondria selectively (36, 49). ULK2 is also a kind of copper-dependent kinase wherein copper ions can release inhibition of ULK2 and enhance its kinase activity (50). It has been reported that when cuproptosis arises, mitophagy can be triggered to maintain mitochondrial homeostasis (51). These findings suggest that ULK2 may be functionally related to autophagy/mitophagy and mitochondrial quality control under copper- or cuproptosis-related stress, although its direct role in PD-related cuproptosis requires further validation. Upregulation of *ULK2* expression could potentially activate autophagy and promote the degradation of α-synuclein, thereby alleviating Parkinson’s disease-like symptoms (52). Copper chaperone for superoxide dismutase (CCS) functions as a cytoplasmic–mitochondrial metallochaperone essential for maintaining intracellular copper homeostasis. Its mechanism involves inserting Cu^+^ ions into the active site of cytoplasmic superoxide dismutase 1 (SOD1), thus enabling its antioxidant capacity (50, 53). Some of the copper chaperone proteins have been demonstrated to be closely involved in regulating the progression of PD through SOD1 activation (54, 55). However, the only currently known chaperone protein capable of directly interacting with and activating human SOD1 is CCS (56). As a chemotactic cytokine, chemotactic C-C ligand 8 (CCL8), alternatively termed monocyte chemotactic protein-2 (MCP-2), plays a crucial role in the recruitment and activation of immune cells (57). Recent studies have also demonstrated its close association with cuproptosis, identifying it as a cuproptosis-related gene (58, 59). Promising evidence suggests *CCL8* participates in neuro-immunoregulation and has been linked to various neurological disorders, including schizophrenia, ischemic stroke, and radiation-induced brain injury (60, 61).

To provide a more comprehensive interpretation of our macro-level findings, we further employed single-cell analysis to investigate cuproptosis-related molecular features at the cellular level within the human substantia nigra, focusing on the four hub DECuAGs and their relationship with PD progression. Our observation revealed that cuproptosis-related scores were increased in PD, particularly evident within ASTs, DaNs, and MGs. Pathologically, the primary characteristic of PD is degeneration of dopaminergic neurons within the substantia nigra. Microglia, the resident immune cells of the brain, are densely distributed in the substantia nigra and striatum regions—both areas affected by PD—thereby linking neuroinflammation with the decline of dopaminergic neurons (62). As the most abundant glial cell population in the brain, astrocytes execute a wide range of critical physiological functions. The loss of regular supportive astrocyte function, coupled with the proliferation of reactive astrocytes exhibiting acquired toxic properties, jointly drives the development and progression of Parkinson’s disease. Dopaminergic neuronal interactions with glial cells, including microglia and astrocytes, maintain the structure and function of parenchymal tissues (63). Increasing evidence suggests that cuproptosis-related processes may be associated with dopaminergic neuronal injury and glial dysfunction, though the underlying mechanisms remain incompletely understood (3, 64). Correspondingly, we observed significant enrichment of FDX1 in these cells, suggesting increased cuproptosis-related molecular alterations in ASTs, DaNs, and MGs during PD. Moreover, opposite expression patterns between FDX1 and the hub DECuAGs were observed in ASTs, DaNs, and MGs, suggesting that these genes may be associated with cell-type-specific cuproptosis-related molecular alterations during PD progression. Based on these findings, we designed *in vivo* expression analyses in the AAV9-A53T mouse model of PD. Through mIF and Western blot analyses in these mouse models, we observed increased cuproptosis-related molecular activity in the PD group compared with the control group, accompanied by reduced expression levels of DLD, ULK2, CCL8, and CCS. This phenomenon was particularly pronounced in DaNs, MGs, and ASTs, consistent with single-cell analysis results.

We further compared the signaling network alterations between PD and control groups using intercellular communication analysis, revealing that MG cells exhibited the greatest alterations. In particular, the NRG signaling pathway played a crucial role, as it was significantly reduced between MGs and ASTs (both were cuproptosis-labeled glial cells) in PD patients. Neuregulins (NRGs) are epidermal growth factor-like ligands that regulate cell growth, survival, and various biological processes (such as anti-inflammation, nervous system development, and their related disorders) through ErbB receptor tyrosine kinases, with NRG1 being relatively well-studied compared to the under-researched NRG2-4 (65). Research has demonstrated that systemic treatment with neurotensin-1β1 in a Parkinson’s disease mouse model protects dopaminergic neurons and improves their function (66). These findings suggest that the significantly reduced NRG signaling pathway in MGs may be associated with PD progression. Taken together, altered NRG–ERBB4-mediated glial communication may represent a candidate molecular bridge linking cuproptosis-associated metabolic alterations to immune and glial dysfunction in PD. However, this interpretation is based on computationally inferred cell–cell communication and should not be considered evidence of a causally proven mediator.

To address the limitation of relying primarily on FDX1 expression to infer cuproptosis-related molecular activity, we added a pharmacological modulation experiment to further examine whether the dysregulation of the four hub DECuAG proteins was linked to pharmacologically modulated cuproptosis-related activity. In PD model mice, TTM treatment, which was used to suppress copper-dependent cuproptosis-related molecular activity, reduced the elevated FDX1 expression and partially restored the decreased levels of DLD, ULK2, CCS, CCL8, and TH. Conversely, Elesclomol-Cu treatment, which was used to enhance cuproptosis-related molecular activity, further increased FDX1 expression and aggravated the reduction of these hub DECuAG proteins and TH. These opposite responses suggest that the downregulation of DLD, ULK2, CCS, and CCL8 is associated with increased cuproptosis-related molecular activity and dopaminergic neurodegenerative changes in the PD model. Moreover, the bidirectional response of FDX1 to TTM and Elesclomol-Cu provides pharmacological support for its use as a responsive cuproptosis-related marker in this study. Nevertheless, this experiment should be interpreted as supportive pharmacological evidence rather than direct proof of gene-specific causality.

There are no specific therapies for Parkinson’s disease despite substantial investment in drug research and development. Clinical management remains primarily symptomatic, with commonly used medications, including dopamine agonists and anticholinergic drugs (9, 65). In this study, we screened the DSigDB database to identify four suitable compounds targeting previously identified hub DECuAGs. 5,7-dimethoxyflavone is a native flavonoid present within numerous plants, exhibiting potent antioxidant and anti-inflammatory activity. Researches indicated that 5,7-dimethoxyflavone can protect neurons from neuroinflammation and oxidative stress damage (67, 68). A study found that plasma levels of α-synuclein in PD patients were significantly correlated with platelet-derived growth factor (PDGF), suggesting the PDGF/PDGFR axis may influence disease progression (69). Crenolanib is a nonselective PDGFR inhibitor and may attenuate PD-associated pathological processes by modulating the PDGF/PDGFR axis (70, 71). The histone deacetylase (HDAC) family may play a significant role in PD progression via protein acetylation, autophagy, and transcriptional repression (72). Trichostatin A (TSA) is a natural HDAC inhibitor produced by Streptomyces bacteria (73). The neuroprotective effect of TSA on dopaminergic neurons is mediated through its ability to induce astrocytes to express glial-derived neurotrophic factor (GDNF) and brain-derived neurotrophic factor (BDNF) (74). Coincidentally, TSA was found to upregulate striatal BDNF expression in the MPTP PD mouse model, thereby protecting dopaminergic neurons (75). Testosterone is the primary male sex hormone, performing multiple vital physiological functions. In the substantia nigra, testosterone was found to be protective against neuronal damage caused by apoptosis and oxidative stress while promoting neuronal generation and elevating brain-derived neurotrophic factor (BDNF), tyrosine hydroxylase (TH), vesicular monoamine transporter (VMAT-2), catechol-O-methyltransferase (COMT), dopamine transporter (DAT), and monoamine oxidase (MAO-A and MAO-B) mRNA expression levels. This study also found that testosterone can improve motor dysfunction and dopaminergic damage in progressive Parkinson’s disease models (76). Through molecular docking analyses and molecular dynamics simulations, we evaluated the predicted binding feasibility and dynamic stability of these candidate compounds with their corresponding target proteins. These findings support them as computationally predicted therapeutic candidates, but their biological effects in PD require further experimental validation.

There are several limitations in this study. First, this study was based primarily on transcriptomic data from public databases. Although supportive validation was performed in an AAV9-A53T mouse model, the expression patterns and clinical potential of the four hub DECuAGs require further verification in larger independent cohorts, clinical samples, and populations with diverse ethnic backgrounds. Second, the single-cell analysis was derived from a single public dataset with a relatively limited sample size and may not fully capture cellular heterogeneity across different disease stages or subtypes; therefore, additional single-cell datasets are needed to improve the generalizability of our conclusions. Third, although cell-type-specific in silico perturbation analyses and pharmacological modulation experiments provided additional support for the functional relevance of *DLD*, *ULK2*, *CCL8*, and *CCS*, this study did not perform direct gene-specific gain- or loss-of-function experiments. Therefore, whether these genes causally regulate cuproptosis-related molecular alterations, α-synuclein aggregation, mitochondrial dysfunction, dopaminergic neuron loss, motor dysfunction, or PD-like phenotypes remains to be determined. Finally, the potential synergistic relationships among the hub genes and the NRG–ERBB4-mediated glial communication inferred by CellChat were based mainly on computational analyses and require further experimental validation.

## Conclusion

In summary, our study systematically identified four cuproptosis-associated hub genes, *DLD*, *ULK2*, *CCL8*, and *CCS*, that were associated with PD through integrated multi-omics data and machine learning methods. Single-cell transcriptomics further elucidated cell-type-specific expression and the involvement of glial cells in altered NRG signaling. *In vivo* validation provided supportive evidence that increased cuproptosis-related molecular activity was accompanied by cell-type-specific downregulation of these genes in dopaminergic neurons, microglia, and astrocytes. In addition, cell-type-specific in silico perturbation analyses provided hypothesis-generating support for the potential functional relevance of these hub DECuAGs, while pharmacological modulation with TTM and Elesclomol-Cu further indicated that FDX1, hub DECuAG protein levels, and TH expression were responsive to modulation of cuproptosis-related molecular activity. Molecular docking and dynamic simulations prioritized several candidate compounds targeting proteins related to these hub genes for further validation. Overall, these findings support a potential link between cuproptosis-associated gene dysregulation and metabolic–immune abnormalities in PD, and provide candidate biomarkers and therapeutic leads for further experimental validation.

## Data Availability

The datasets presented in this study can be found in online repositories. The names of the repository/repositories and accession number(s) can be found in the article/**Supplementary Material**.
