# Supplementary material for: Cuproptosis-associated genes DLD, ULK2, CCL8, and CCS link metabolic and immunological dysfunction in Parkinson’s disease: a multi-omics study
Source: Front Immunol. 2026 Sep 16;17:1813166. doi: 10.3389/fimmu.2026.1813166 (PMC13623572; doi:10.3389/fimmu.2026.1813166)
Supplement: Supplementary file 1 [file DataSheet1.docx]

Figure S1. Enrichment analysis of DEGs between PD and the controls. (A) Enrichment results color-coded by statistical significance in the Metascape database. (B) Bar plot of GO analysis results in DECuAGs. (C) Bubble plot of GO analysis results in DECuAGs. (D) Bar plot of enriched KEGG pathways in DECuAGs. (E) Bubble plot of enriched KEGG pathways in DECuAGs.

Figure S2. Enrichment analysis of DECuAGs between PD and the controls. (A) Circle plot of GO analysis results in DECuAGs. (B) Bubble plot of GO analysis results in DECuAGs. (C) Bar plot of DO analysis results in DECuAGs. (D) Disease enrichment analysis results from the Metascape database.

Figure S3. GSEA and ssGSEA analysis for hub DECuAGs between PD and the controls. GO analysis revealed significant biological functions in the high and low expressions of DLD (A), ULK2 (B), CCL8 (C), and CCS (D). KEGG pathway analysis revealed significant biological processes in the high and low expressions of DLD (E), ULK2 (F), and CCL8 (G). (H) Heatmap displays Spearman's correlation coefficients between the four genes and HALLMARK enrichment scores.

Figure S4. Immunological characteristics in PD and their correlation with hub DECuAGs. (A) Differences in immune-infiltrating cells between PD and control samples. (B) Boxplot illustrating variations in the immune microenvironment between PD and control samples. (C) Correlation analysis between the 22 immune-infiltrating cells in PD samples. (D) Correlation analysis of hub DECuAGs expression with immune cell abundance in PD samples. (E) Scatter plots demonstrating a negative correlation between ULK2 expression and macrophage infiltration. (F) Integrated correlation-network analysis of gene-immune cell interactions in PD. Nodes represent cell types and genes, while edges denote interactions.

Figure S5. Single-cell analysis. (A) Quality control of the GSE243639 dataset. (B) Scatter plot of the correlations of sequencing depth. (C) Volcano plot of the top 3000 most variable genes in normalized data. (D) Elbow plot of the principal components in PCA. (E) JackStraw plot of the principal components in PCA. (F) The feature genes of each principal component. (G) Heatmap of the PCA feature genes. (H) Heatmap of top marker genes expression across 8 cell clusters. (I) Violin plots comparing cuproptosis activity across distinct cell types between PD and the controls. (J) Violin plots comparing expression levels of hub DECuAGs between PD and the controls. (K) Volcano plots of hub DECuAGs in distinct cell types between PD and the controls. (L) Half-violin plots comparing the expression levels of hub DECuAGs across distinct cell types between PD and the controls.

Figure S6. Cell-type-specific in silico perturbation analysis of *DLD* in dopaminergic neurons. (A) GO and KEGG enrichment analysis of genes responsive to simulated *DLD* knockout in control DaNs. (B) GO and KEGG enrichment analysis of genes responsive to simulated *DLD* knockout in PD DaNs. (C) GO and KEGG enrichment analysis of genes responsive to simulated *DLD* overexpression in control DaNs. (D) GO and KEGG enrichment analysis of genes responsive to simulated *DLD* overexpression in PD DaNs. The top enriched biological process (BP), cellular component (CC), molecular function (MF), and KEGG terms are ranked by −log10(P value).

Figure S7. Cell-type-specific in silico perturbation analysis of *CCS* in astrocytes. (A) GO and KEGG enrichment analysis of genes responsive to simulated *CCS* knockout in control ASTs. (B) GO and KEGG enrichment analysis of genes responsive to simulated *CCS* knockout in PD ASTs. (C) GO and KEGG enrichment analysis of genes responsive to simulated *CCS* overexpression in control ASTs. (D) GO and KEGG enrichment analysis of genes responsive to simulated *CCS* overexpression in PD ASTs. The top enriched biological process (BP), cellular component (CC), molecular function (MF), and KEGG terms are ranked by −log10(P value).

Figure S8. Cell-type-specific in silico perturbation analysis of *ULK2* in microglia. (A) GO and KEGG enrichment analysis of genes responsive to simulated *ULK2* knockout in control MGs. (B) GO and KEGG enrichment analysis of genes responsive to simulated *ULK2* knockout in PD MGs. (C) GO and KEGG enrichment analysis of genes responsive to simulated *ULK2* overexpression in control MGs. (D) GO and KEGG enrichment analysis of genes responsive to simulated *ULK2* overexpression in PD MGs. The top enriched biological process (BP), cellular component (CC), molecular function (MF), and KEGG terms are ranked by −log10(P value).

Figure S9. Cell-type-specific in silico perturbation analysis of *CCL8* in microglia. (A) GO and KEGG enrichment analysis of genes responsive to simulated *CCL8* knockout in control MGs. (B) GO and KEGG enrichment analysis of genes responsive to simulated *CCL8* knockout in PD MGs. (C) GO and KEGG enrichment analysis of genes responsive to simulated *CCL8* overexpression in control MGs. (D) GO and KEGG enrichment analysis of genes responsive to simulated *CCL8* overexpression in PD MGs. The top enriched biological process (BP), cellular component (CC), molecular function (MF), and KEGG terms are ranked by −log10(P value).

Figure S10. Cell-cell communication analysis. (A) Heatmaps of the number and strength of intracellular communication networks in PD and the controls. (B) Bubble plot displaying the enriched pathways, ligands, and receptors involved in cell-cell communication among PD and the controls. (C) Circle plots illustrating the strength of the NRG signaling network in PD and the controls. (D) Heatmaps illustrating the roles of each cell type in the NRG signaling pathway. (E) The contribution of the NRG signaling pathway in PD and the controls.
