# Supplementary material for: Cuproptosis-associated genes DLD, ULK2, CCL8, and CCS link metabolic and immunological dysfunction in Parkinson’s disease: a multi-omics study
Source: Front Immunol. 2026 Sep 16;17:1813166. doi: 10.3389/fimmu.2026.1813166 (PMC13623572; doi:10.3389/fimmu.2026.1813166)
Supplement: Supplementary file 2 [file Table1.docx]

Supplementary Table 1. The specific information of datasets in this study.

| **Dataset** | **Platform** | **Normal samples** | **PD samples** | **Type** |
| --- | --- | --- | --- | --- |
| GSE28894 | GPL6104 (Illumina humanRef-8 v2.0 expression beadchip) | 55 | 59 | Messenger RNAs (brain) |
| GSE26927 | GPL6255 (Illumina humanRef-8 v2.0 expression beadchip) | 12 | 8 | Messenger RNAs (brain) |
| GSE20186 | GPL6947 (Illumina HumanHT-12 V3.0 expression beadchip) | 18 | 18 | Messenger RNAs (brain) |
| GSE42966 | GPL413 (Agilent-014850 Whole Human Genome Microarray 4x44K G4112F) | 6 | 9 | Messenger RNAs (brain) |
| GSE49126 | GPL4133 (Agilent-014850 Whole Human Genome Microarray 4x44K G4112F) | 20 | 30 | Messenger RNAs (blood) |
| GSE243639 | GPL24676 (Illumina NovaSeq 6000) | 14 | 15 | Single-cell RNA (brain) |

PD, Parkinson's disease
